# Optimization of Calibration Strategies for the Quantification of Volatile Compounds in Virgin Olive Oil

**DOI:** 10.3390/foods14193439

**Published:** 2025-10-08

**Authors:** Enrique J. Díaz-Montaña, María Barbero-López, Ramón Aparicio-Ruiz, Diego L. García-González, María T. Morales

**Affiliations:** 1 Department of Analytical Chemistry, Faculty of Pharmacy, University of Seville, Prof. García González, 2, 41012 Seville, Spain; aparicioruiz@us.es; 2 Instituto de la Grasa (IG), CSIC, Edificio 46, Ctra. de Utrera, Km. 1, 41013 Seville, Spain; mbarbero@ig.csic.es (M.B.-L.); dlgarcia@ig.csic.es (D.L.G.-G.)

**Keywords:** virgin olive oil, volatile compounds, external matrix-matched standard calibration, internal standard calibration, standard addition calibration

## Abstract

The quantification of volatile compounds in virgin olive oil poses several analytical challenges due to the existence of different concentrations, chemical families, and the possible matrix effect. Accurate quantification, using adequate methodological calibration and statistical procedures, is essential for obtaining reliable results. The aim of this work was to develop and validate an analytical–statistical approach for the quantification of volatile compounds in virgin olive oil. Therefore, several analytical parameters were determined for four calibrations. The ordinary least square (OLS) linear adjustment was selected over the weighted least square due to the homoscedasticity of the variable errors. Additionally, standard addition (AC) and AC with an internal standard (IS) exhibited greater variability, whereas external matrix-matched calibration (EC) was identified as the most reliable approach for quantifying volatile compounds in virgin olive oil. The employment of an IS did not improve the performance of the method in any case. Thus, based on the statistical results, the OLS linear adjustment with EC was selected as the best statistical–analytical approach for quantifying volatiles in olive oil matrices. The volatiles of nine virgin olive oil samples were quantified, applying different methodological calibrations, and no differences were detected, underscoring EC as a superior alternative.

## 1. Introduction

Virgin olive oil (VOO) is made up of different components that are classified into major and minor fractions. The minor fraction accounts for approximately 2%, and it is constituted by a wide number of compounds from different chemical families, such as sterols, terpenic alcohols, aliphatic alcohols, tocopherols, hydrocarbons, phenolic compounds, pigments, and volatile compounds, among others [1,2]. The study and analysis of these minor compounds serve as a chemical fingerprint of virgin olive oil (VOO) for the authentication, characterization, and identification of the geographical origins and olive variety, among others [3,4,5].

Volatile compounds within this minor fraction are particularly significant because they are the main contributors to the VOO’s characteristic aroma [4]. The official classification of VOO into categories (extra virgin, virgin, or lampante) is performed following EU regulations, which rely on specific physico-chemical parameters and a sensory evaluation [6,7]. Extra virgin olive oil (EVOO) is the highest quality grade, distinguished by the complete absence of sensory defects; virgin olive oil (VOO) is of slightly lower quality, as it presents low intensity sensory defects; lastly, lampante virgin olive oil, by contrast, exhibits pronounced sensory defects, being unsuitable for direct human consumption and needing to undergo a refining process. The sensory evaluation is a slow and expensive procedure, but is crucial because, depending on the result, virgin olive oil is classified into different categories which impact its final economic value [6,8]. Even though the sensory analysis is related to the volatile composition of VOO, it cannot be directly correlated with the concentration of certain volatile compounds due to the complexity of the virgin olive oil matrix and the involvement of the neurophysiological system. However, there are numerous studies that relate the presence and concentration of the determined compounds or compound mixture to sensory attributes and defects and their intensity [9,10,11]. Consequently, analytical methods based on the identification and quantification of volatiles are commonly used to support the panel test and provide a chemical fingerprint for applications like the discrimination of Protected Designations of Origin (PDO) or authentication [5,12].

The analysis of volatile compounds involves some complexity because the analytes are present in low and different concentration ranges, have different chemical characteristics, and are in a complex oily matrix. The current methodologies typically involve a preconcentration step, such as Solid Phase Microextraction (SPME) or Dynamic Head Space (DHS), followed by Gas Chromatography (GC) coupled with a detector (e.g., flame ionization detector (FID) or mass spectrometry (MS)), among other techniques or detectors [13,14]. The accurate quantification of these analytes is essential for reliable results, generally employing methods like external standard calibration with an internal standard (ES with IS) or semi-quantification via internal standard calibration (IC) [12,13,15,16,17].

External matrix-matched standard calibration (EC) is the most employed calibration procedure when working with oily matrices. The reference material (RM) is the same analyte to be quantified, and it is measured separately from the samples but is prepared in a matrix as similar as possible to that of the real samples. Two or more standards at different concentrations are measured and related to their signals. Once the calibration curve is performed, the signal of the sample can be interpolated and the unknown analyte concentration can be determined. EC can be used as long as there are no differences between the standard and the sample matrices [18,19]. This calibration is the easiest one because, with one calibration curve, several samples can be interpolated and quantified.

When dealing with oily matrices, the choice of the calibration procedure is critical and requires careful validation. While external matrix-matched standard calibration (EC) is a common, straightforward approach, it assumes no significant difference between the standard’s simulated matrix and the actual sample matrix [18,19]. Conversely, standard addition calibration (AC) is more time-consuming, as it requires a calibration line for each sample, but it is necessary when a strong matrix effect interferes with the analyte signal [20,21]. The use of internal standards (IS), often for instrument response correction, can also be utilized for semi-quantification purposes (IC) [18,22,23]. Despite the availability of these techniques, many published papers quantify VOO volatiles using only the EC with IS methodology, without considering or evaluating other calibration procedures [12].

Despite the necessity of method validation to ensure the high analytical quality and trueness of the results based on parameters like sensitivity, accuracy, and precision [24,25,26,27], a critical gap remains in the literature. The published methods for VOO volatile determination are not always fully validated, and, crucially, they often fail to explicitly confirm the absence or presence of the matrix effect when selecting a calibration strategy.

Therefore, the aim of this work was to develop and validate an analytical–statistical approach for the quantification of volatile compounds in virgin olive oil by evaluating the linearity, limit of detection and quantification (LOD and LOQ), accuracy, precision, and matrix effect, among other parameters, across four distinct calibrations procedures.

## 2. Materials and Methods

### 2.1. Chemicals

All the reagents employed were of pure analytical grade. Ethyl acetate, (*Z*)-3-hexenyl acetate, 1-octen-3-ol, (*E*)-2-pentenal, (*E*)-2-hexenol, 6-methyl-5-hepten-2-one, and isobutyl acetate (internal standard) were purchased from Merck (Darmstadt, Germany). Pentanal, hexanal, hexyl acetate, hexan-1-ol, (*Z*)-3-hexenol, (*E*)-2-hexenal, and acetic acid were from Panreac (Barcelona, Spain).

### 2.2. Samples

Due to the existing difference between extra virgin olive oil (EVOO), virgin olive oil (VOO), and lampante virgin olive oil, three samples of each category were selected. The extra virgin olive oils (EVOOs) were from monovarietal Picual, Arbequina, and Coratina varieties; the virgin olive oils (VOOs) were from the Hojiblanca variety, Coratina variety, and a mixture of olive varieties, all of them (EVOOs and VOOs) from the 2022/2023 season. Since lampante oil cannot be marketed before refining, three EVOOs (Picuda, Arbequina, and Cornicabra varieties) from the 2016/2017 season were purchased in 2017 and stored under conditions simulating commercial storage until these samples reached the lampante criteria, after which they were stored under freezing [6].

Refined olive oil, provided by Acesur (Seville, Spain), was used as an oil matrix to prepare the standards. Prior to use, the oil was analyzed to confirm the absence of volatile compounds.

### 2.3. Quality Parameters and Sensory Assessment

The quality parameters and sensory assessment are established by European Union regulations and include the median of defect, median of fruity sensory attribute, acidity index, peroxide value, UV absorbance (K_232_, K_268_, or K_270_, ΔK), and fatty acid ethyl esters [6]. All the reagents employed were of analytical reagent grade. Methanol, hexane, and ethyl acetate were purchased from Panreac (Barcelona, Catalonia, Spain), and p-hydrophenylacetic acid, o-coumaric acid, orthophosphoric acid, and acetonitrile were purchased from Merck (Darmstadt, DE, Germany).

### 2.4. Volatile Compound Analysis

Volatile compounds were analyzed by Dynamic Head Space (HT3 Dynamic System, Teledyne Tekmar, Mason, OH, USA)—Gas Chromatography (Varian 3900, Palo Alto, CA, USA) coupled to a flame ionization detector (DHS-GC-FID). For this procedure, 1.5 g of sample was placed into a 20 mL glass vial and tightly sealed with a silicone/polytetrafluoroethylene (PTFE) septum. Firstly, the sample was pre-heated for 18 min at 40 °C and mixed for 15 min. Secondly, the volatile compounds present in the head space were moved to the adsorbent trap (Tenax TA^TM^, Buchem B.V., Apeldoorn, The Netherlands) with helium as portable gas with a flow rate of 5 mL/min. The volatiles adsorbed by the trap were thermally desorbed into the hot injection port of the GC for 5 min at 260 °C with a split mode 7:1. The carrier gas was hydrogen at a flow rate of 1.5 mL/min. The oven temperature was held at 35 °C for 10 min and then programmed to rise at 3 °C/min to a final temperature of 200 °C for 1 min. The column was a silica TRB-WAX (60 m × 0.25 nm × 0.25 µm; Teknokroma, Barcelona, Spain). The temperature of the flame ionization detector (FID) was set at 280 °C. The chromatographic signals were recorded and processed with Star Chromatography Workstation, System Control version 6 (Palo Alto, CA, USA).

The analysis of the samples and calibration curves were conducted in triplicate.

#### 2.4.1. External Matrix-Matched Standard Calibration (EC)

A calibration curve per compound, in a refined olive oil matrix, was carried out with concentrations between 0.1 and 10.5 mg/kg, with a total of fourteen points per curve with an interval of 0.8 mg/kg between them. This concentration range was selected based on the volatile concentration determined in previous studies [4,28]. Additionally, two higher concentrations (15 and 25 mg/kg) were also measured to ensure that, if any compound showed a concentration higher than 10.5 mg/kg in a sample, the GC-FID response still linear.

#### 2.4.2. External Matrix-Matched Standard with Internal Standard Calibration (EC with IS)

For EC with IS, 0.100 g of a solution of 1000 mg/kg of the internal standard (isobutyl acetate), prepared in refined olive oil, was added to each point of the EC curve and to the samples analyzed.

#### 2.4.3. Standard Addition Calibration (AC)

The concentration range for AC was the same as that in EC, from 0.1 to 10.5 mg/kg with an interval of 0.8 mg/kg.

#### 2.4.4. Standard Addition with Internal Standard Calibration (AC with IS)

For AC with IS, 0.100 g of a solution of 1000 mg/kg of isobutyl acetate in refined olive oil was added to each point of the AC curve.

### 2.5. Analytical–Statistical Approach

#### 2.5.1. Linearity

The initial step in method development and validation is to ensure that the theoretical model fits on the calibration curve, generally linear when a short concentration range is employed, but quadratic when working with a concentration range of 2–3 orders of magnitude. A linear model can be adjusted by ordinary least squares regression (OLS) or weighted least squares regression (WLS); the choice of one or the other will depend on the homoscedasticity of the variable errors (variances). Homoscedasticity can be evaluated using the residual plot, a standard deviation graph, and the *F* test [29]. The model selection (OLS or WLS) was performed with the calibration curves obtained by external matrix-matched standard calibration.

The *F* test was applied as a statistical tool to confirm homoscedasticity. In this test (Equation (1)), the value of *F* is calculated ( $F_{cal}$ ) comparing the highest ( $s_{max}^{2}$ ) and lowest ( $s_{min}^{2}$ ) variances shown by the points of the calibration curve, and it is compared to the tabulated *F* ( $F_{tab}$ ), with α = 0.05 and N-1 as degrees of freedom for the numerator (v_1_) and denominator (v_2_), where N is the number of curve replicates (each curve was analyzed in triplicate) [30].

(1) $$F_{cal}=\frac{s_{max}^{2}}{s_{min}^{2}}$$

Once the homoscedasticity was proven, different *t* tests were performed (i) to confirm the linearity of the analytical curve by the comparison of the slope (b) and intercept (a), with their standard deviations ( $s_{b}$ and $s_{a}$ ) (Equations (2) and (3)); and (ii) to test the adjustment of the linear model to the calibration curve (Equation (4)) [29,31]. The calculated *t* ( $t_{cal}$ ) was compared to the tabulated *t* ( $t_{tab}$ ) with α = 0.05 and N_x_-2 degrees of freedom (N_x_ being the number of points of the calibration curve). As well as for *F* tests, the $t_{tab}$ is the same for all the compounds and *t* tests conducted, being 2.18. The *n* refers to the number of points in the calibration curve, and *r* refers to the correlation coefficient. If $t_{cal}\geq t_{tab}$ , the linearity of the concentration range cannot be discarded. As for the linearity adjustment, if the $t_{cal}$ for the slope and intercept is greater than the tabulated one, the parameters cannot be discarded due to the existence of statistically significant difference (statistical hypothesis accepted).

(2) $$t_{cal,\;b}=\frac{b}{s_{b}}$$

(3) $$t_{cal,\;a}=\frac{a}{s_{a}}$$

(4) $$t_{cal}=\left|r\right|\sqrt{\frac{n-2}{1-r^{2}}}$$

As mentioned before, when working in a wide concentration range, the calibration curve may exhibit quadratic behavior. Thus, to confirm the linearity—and not merely avoid discarding it—a Mandel test needs to be performed. The Mandel test is a comparison between the quadratic and linear model following Equation (5).

(5) $$F_{Mandel}=\frac{\frac{{SS}_{linear}-{SS}_{quadratic}}{{df}_{linear}-{df}_{quadratic}}}{\frac{{SS}_{quadratic}}{{df}_{quadratic}}}$$

where *SS* is the residual sum of the square of the linear/quadratic model and *df* is the degrees of freedom of the linear/quadratic model. If *F_Mandel_* is lower than the tabulated F (*F_tab_*), linearity can be confirmed.

Additionally, other linear parameters were determined. These parameters encompass the presence/absence of outliers, the determination coefficient (*R*^2^), and the relative standard deviation of the slope (*RSD_b_*; ${RSD}_{b}\left(\%\right)=\frac{S_{b}}{b}*100$ , *S_b_* being the standard deviation of the slope and *b* being the slope).

#### 2.5.2. Sensitivity

The sensitivity was also evaluated in this topic due to the link between linearity and sensitivity, as the sensitivity is understood as the slope of the analytical curves.

#### 2.5.3. Accuracy

Accuracy, expressed as the relative recovery, was determined as the concentration obtained from spiked matrices after sample concentration correction (in AC and AC with IS calibrations), divided by the spiked concentration and multiplied by 100%. It was performed at 0.1, 1, and 10 mg/kg concentration levels in triplicate.

#### 2.5.4. Limit of Detection (LOD) and Limit of Quantification (LOQ)

Once the linearity and accuracy were evaluated, limit of detection (LOD) and limit of quantification (LOQ) were determined. The LOD and LOQ were calculated as three and ten times the ratio signal/noise, respectively.

#### 2.5.5. Precision

Precision is considered as the similarity between the replicates of independent analysis. In this study, repeatability and intermediate precision were evaluated. Precision was calculated as the coefficient of variation (*CV*; $CV\left(\%\right)=\frac{S}{\bar{X}}*100$ , *S* being the standard deviation and $\bar{X}$ being the mean of the analysis results). The precision study was performed in nine replicates (nonuplicate); the repeatability was performed in the same day, by the same operator, whereas the intermediate precision was conducted on three different days by two operators. Additionally, as for accuracy, three concentration levels were tested (0.1, 1, and 10 mg/kg).

#### 2.5.6. Matrix Effect

The existence or not of a matrix effect can be performed graphically, comparing the plotted curves, but it should be performed by the application of statistical analysis. There are two ways of demonstrating the existence of a matrix effect: (i) comparing the slope ratio of two of the four slopes (EC–AC ratio or EC with IS–AC with IS ratio) with 1 by a *t* test—if there is no significant difference between the ratio and 1, that means that there are no differences in the slopes, thus there is no matrix effect; or (ii) a more accurate way, and the one used in this study, employing the *t* test, the expanded uncertainty (U, with the coverage factor (*k*), Equation (6)) considering the slope ratio and the standard deviation of the two curves being compared (Equations (7)–(9)). The coverage factor “produces an interval about the measurement result *y* that may be expected to encompass a large, specified fraction p of the distribution of values that could reasonably be attributed to the measurand Y”; for calculating the level of confidence (Z), a normal distribution and the standard deviation (s) are needed (Equation (7)) [32]. Normally, for having an approximately 95% level of confidence, the coverage factor is 2 [32]. The coverage factor is employed to determine the expanded uncertainty (U) [33]. As explained before, the slope-ratio can be around 1 (if there is no matrix effect); thus, to be able to have a significant difference between them, the statistical analysis should consider a standard deviation ±1. Therefore, with all these considerations, the expanded uncertainty, which is the combined standard deviation (S_R_) multiplied by the coverage factor (*k* = 2), is, for this statistical study, ±2. Once the expanded uncertainty is determined, the next step will be to calculate the $t_{cal}$ and, finally, to compare the U and $t_{cal}$ . Equation (7) describes the relation (R) between the slopes ( $b_{1}$ and $b_{2}$ ) of the two curves being compared, and it should be one or more. Furthermore, Equation (8) is used to calculate the combined variance ( $S_{R}^{2}$ ), by the employment of *R*, of the slopes and the standard deviations of the slopes ( $s_{b1}^{2}$ and $s_{b2}^{2}$ ). Finally, the *t* can be calculated (Equation (9)) with the data obtained before. When the calculated *t* is equal to or higher than the expanded uncertainty (*U* = 2), there is a significant difference between both analytical curves, meaning the existence of a matrix effect. In contrast, if t<U=2 , the matrix effect can be discarded, and other considerations (economical, time-consuming, among others) need to be considered for choosing the methodological calibration.

(6) $$k=Z*s;U=k*S_{R}$$

(7) $$R=\frac{b_{1}}{b_{2}}\geq 1$$

(8) $$S_{R}^{2}=R^{2}\left(\frac{s_{b1}^{2}}{b_{1}^{2}}+\frac{s_{b2}^{2}}{b_{2}^{2}}\right)$$

(9) $$t=\frac{R-1}{S_{R}}$$

### 2.6. Statistical Analysis

The whole set of data was imported to Excel 2016 (Microsoft Corp., Redmond, WA, USA) from the instrument software. Excel 2016 was employed to carry out the univariate statistical treatment, including the resolution of the equations. Statistica release 8.0 (StatSoft, Tulsa, OK, USA) was used to perform the data analysis, including the principal component analysis (PCA).

## 3. Result and Discussion

### 3.1. Samples Characterization

The samples were classified according to the EU regulations [6]. The analysis showed that three samples were of the extra virgin category, three were of the virgin category, and, finally, after a long storage, the other three samples reached the lampante virgin olive oil category (Table 1).

### 3.2. Analytical–Statistical Method Validation

#### 3.2.1. Linearity

**Figure 1:**
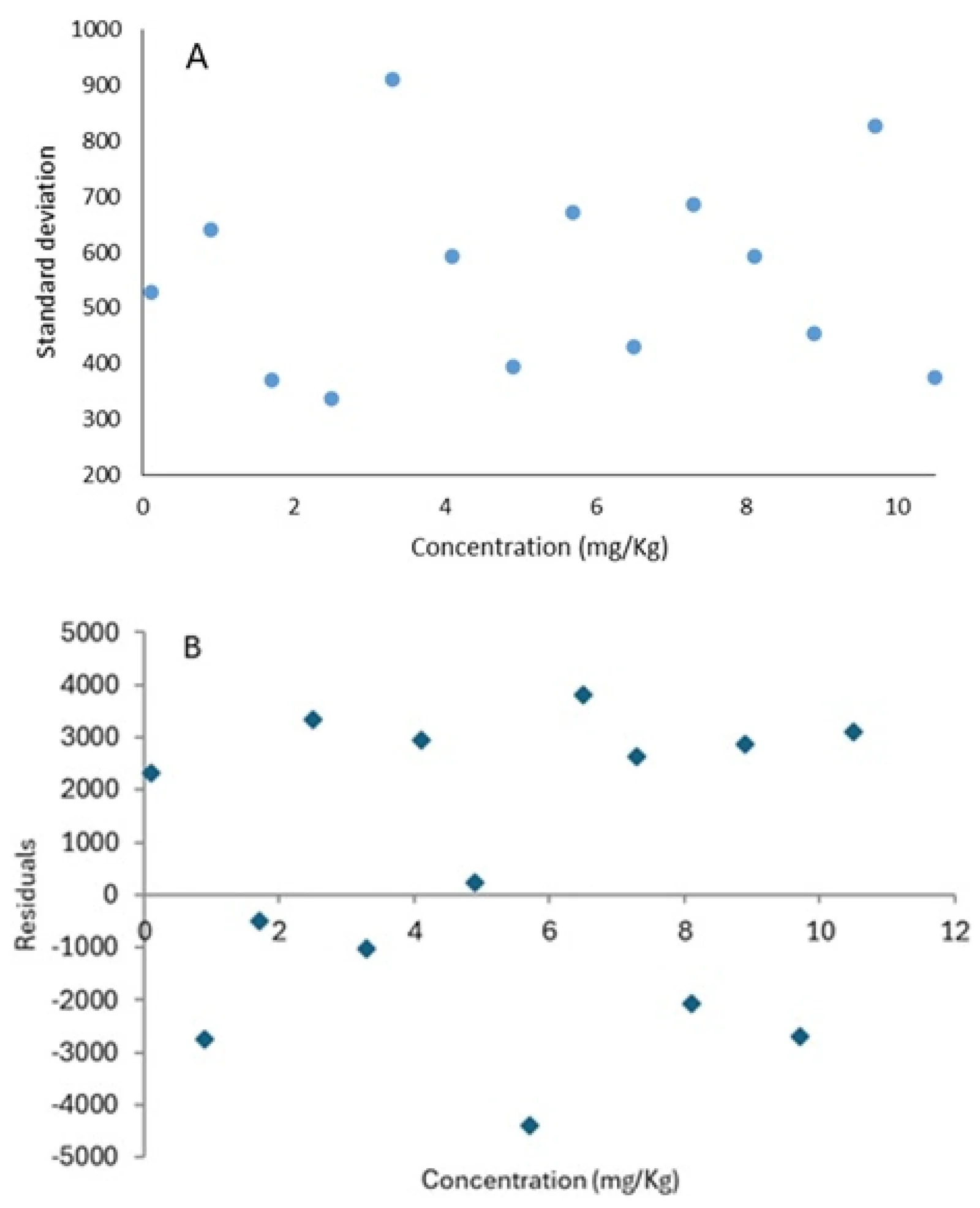
Standard deviation (**A**) and residual plot (**B**) as a function of the concentration (mg/kg) for hexanal.

Firstly, the homoscedasticity of the data was evaluated using both the standard deviation graph and the residual plots. In the case of hexanal, the standard deviation graph (Figure 1A) showed no evident trend, indicating a constant variance. Additionally, the residual plot (Figure 1B) was assessed to verify the three key assumptions of residual analysis—independence, normality, and homogeneity of variance—all of which were satisfied, further supporting the assumption of homoscedasticity. This graphical evaluation was conducted for the 13 studied compounds, and no evidence of heteroscedasticity was detected in any case. To complement the visual evaluation and reduce the associated potential bias, an *F*-test was performed (Table 2). This statistical test was based on 14-point calibration curves measured in triplicate (N = 3), with a critical *F* value (*F_tab_*) of 19 applied uniformly across all compounds and analyses. The calculated *F* values (*F_cal_*) ranged from 2.53 for hexyl acetate to 11.94 for (*E*)-2-hexenal, all of which were below the critical threshold (*F_cal_* < *F_tab_*). Thus, heteroscedasticity was statistically discarded. Given the confirmed homoscedasticity, the ordinary least squares (OLS) model was selected, and the weighted least squares (WLS) approach was not required. Although some authors prefer to apply WLS preemptively when switching matrices to account for possible variance inconsistencies [34], the current analysis demonstrates that such a correction is unnecessary in this case.

Once the linear model adjustment was selected, the linearity was tested through *t* tests and Mandel’s test, where Mandel’s test compares the quadratic and linear models, confirming the adequate one. As shown in Table 2, the three $t_{cal}$ are above the tabulated *t* ( $t_{tab}=2.18$ ), ensuring that the slope and intercept need to be employed. The intercept can be mainly discarded when employing external standard calibration, but, due to the employment of matrix-matched calibration and standard addition, it needs to be considered. Acetic acid $t_{cal,a}$ was the only value in the limit of $t_{tab}$ ; even though it is accepted as a significant difference, this small difference with the tabulated value is due to the variability of the signal of this compound. As can be seen in Table S1, acetic acid did not exhibit satisfactory linearity with respect to the different parameters in any of the methodological calibrations.

Additionally, to confirm the linearity of the calibration concentration range, the Mandel test (*F_Mandel_*) was performed. Table 2 shows that, for all the chemical compounds, the *F_Mandel_* < *F_tab_* ( $F_{tab}=19$ ), thus indicating that the quadratic approach can be discarded and confirming the linear behavior of the calibration curve. Therefore, based on the statistical analysis, the OLS linear adjustment was confirmed and the linearity of the model was confirmed through the *t* test and Mandel’s test.

Additionally, different parameters (calibration curve equation, *R*^2^, *RSD_b_*, and sensitivity) were evaluated for the four methodological calibrations (Table S1). For the coefficient of determination (*R*^2^), even though it should not be the only parameter to base our analysis on, it is interesting to have a preliminary idea of the linear fitting. Three calibrations showed values over 0.98, with no significant (*p* < 0.05) difference between them, highlighting the good linear modeling of the different calibrations. However, the EC with IS showed in all cases (except (*Z*)-3-hexenyl acetate) a lower *R*^2^, being two calibrations below 0.98. In certain chemicals, such as hexanal or (*E*)-2-hexenal, these differences are significant (*p* < 0.05), shedding light on the fact that the employment of an internal standard could not be beneficial for the quantification of volatiles in these kinds of matrices; however, the rest of the parameters need to be evaluated to reach this conclusion. Additionally, as in the study of linearity, acetic acid showed the worst *R*^2^ in comparison to the rest of the chemicals, but the EC with IS still showed the lowest value (0.9270).

**Figure 2:**
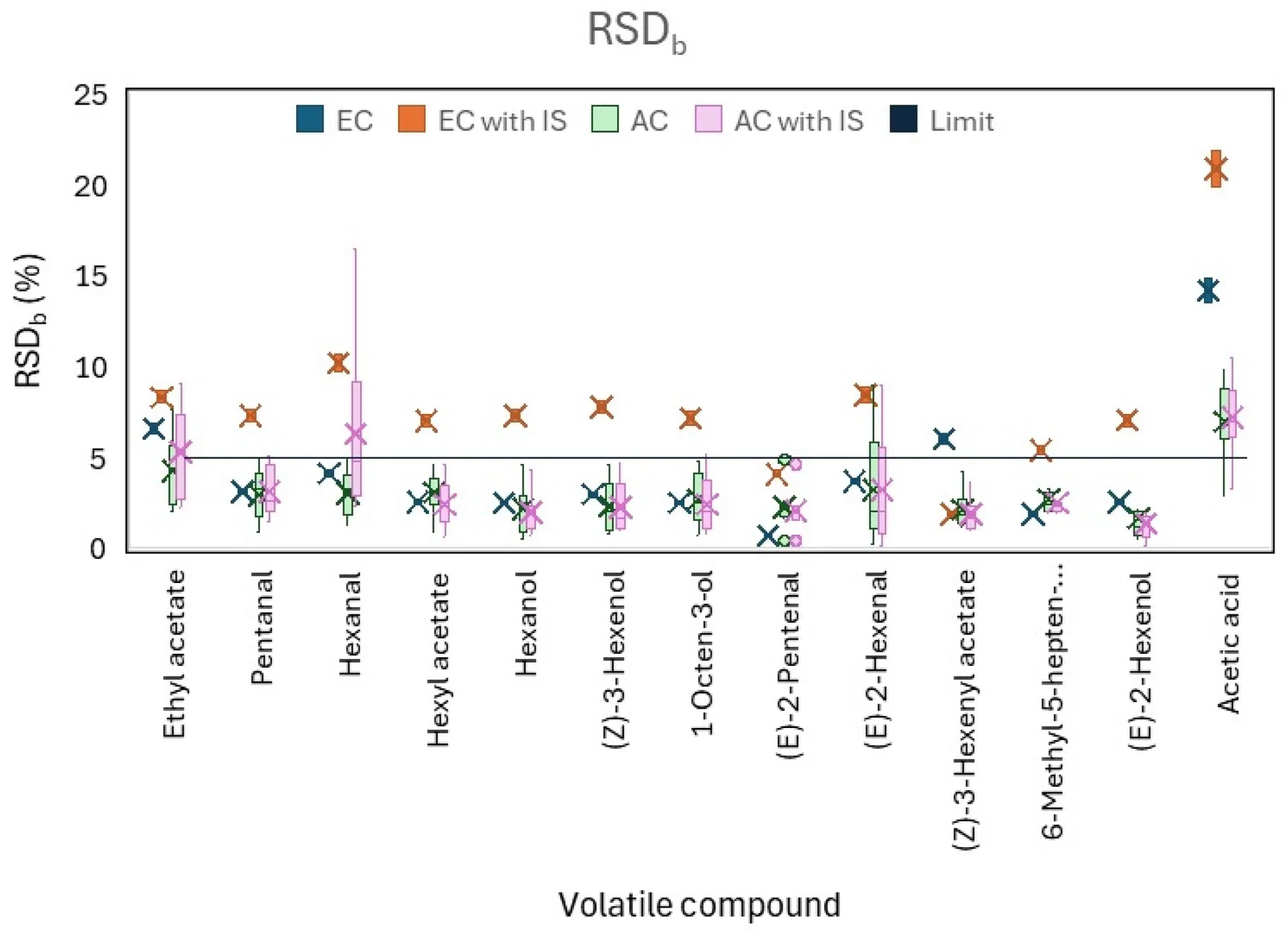
Box and whisker plots of *RSD_b_* of the four methodological calibrations (EC: blue, EC with IS: orange, AC: green, and AC with IS: sky blue) for each compound. The cross indicates the mean, the box is the 95% quartile, and the bars go from minimum to maximum if they are out of the box. In the case of RSD, the line corresponds to the 5% limit established.

Figure 2 shows the *RSD_b_* parameters for the four methodological calibrations, considering AC and AC with IS as the mean of the nine (one per sample) different analytical curves obtained. The *RSD_b_* was below 5% in the standard addition calibration for all the chemicals (except acetic acid); the other three calibrations went over this limit for at least two chemicals. The external matrix-matched calibration went over the 5% for ethyl acetate (6.56%), (*Z*)-3-hexenyl acetate (6.00%), and acetic acid (14.18%), while the rest of compounds were below, ranging from 0.67% ((*E*)-2-pentenal) to 3.75% (hexanal), but it presented the lowest associated errors bars. The *RSD_b_* of EC with IS was over 5% in all the chemicals, except for (*E*)-2-pentenal, with 4.08%. This calibration did also present a low associated error, but higher than with EC. As mentioned, in the case of standard addition calibration, all the volatile compounds, except acetic acid (6.96%), showed an *RSD_b_* below 5%, but with a wider error. Lastly, the AC with IS went over the 5% limit for ethyl acetate (5.31%), hexanal (6.29%), and acetic acid (7.19%). Additionally, AC with IS presented the highest error associated with the *RSD_b_*. Thus, the employment of an internal standard did not improve the *RSD_b_* parameter; the standard addition calibrations (AC and AC with IS) showed wider errors due to the increase in the number of calibration analyses (one per sample in triplicate) in comparison to the external standard calibrations (one calibration curve in triplicate). Additionally, it is remarkable that for all calibrations the acetic acid surpassed the 5% limit, highlighting the difficultness of quantifying this volatile compound by using this kind of method.

#### 3.2.2. Sensitivity

**Figure 3:**
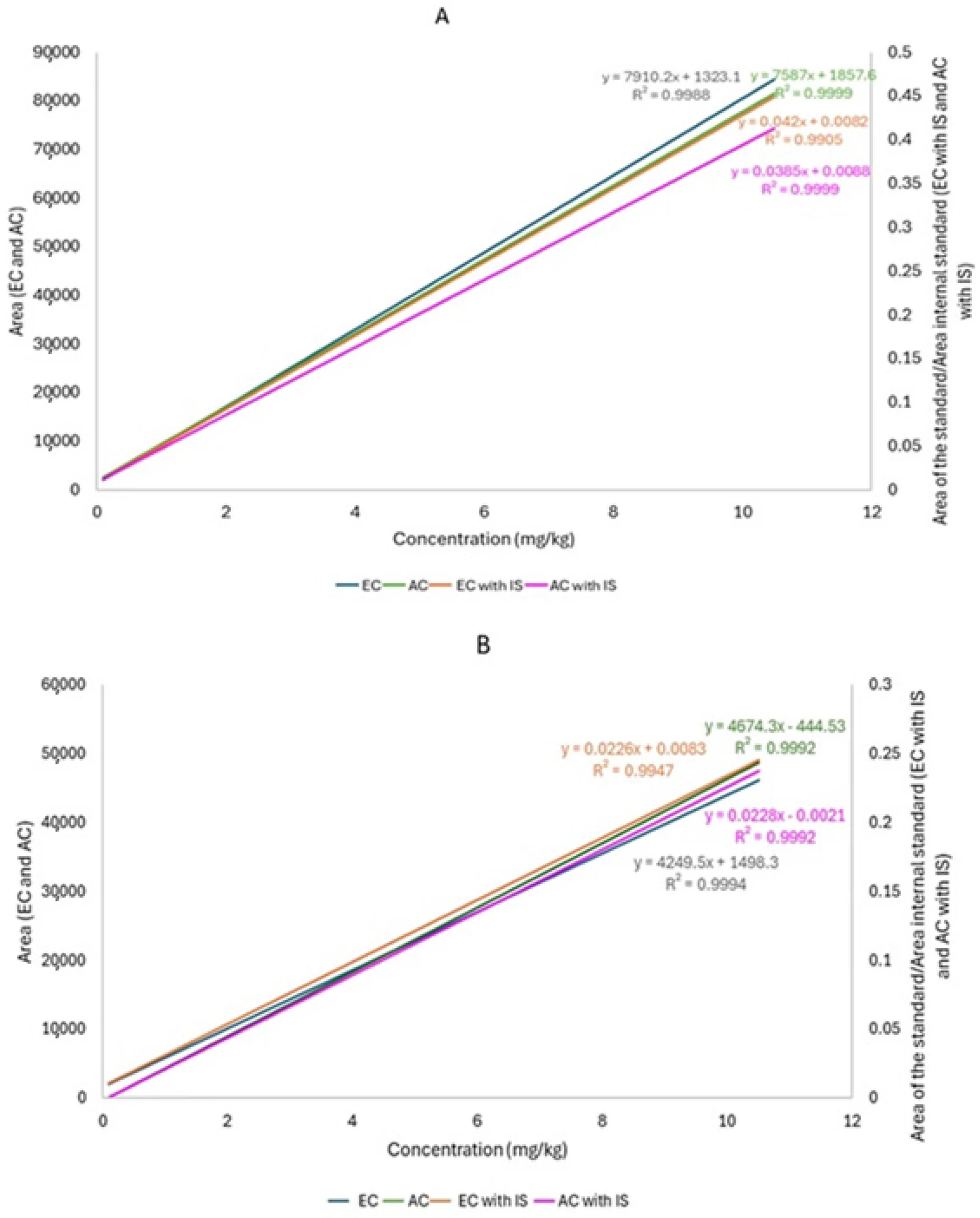
The four calibration curves (EC (blue), EC with IS (orange), AC (green), and AC with IS (pink)) with the curve equation and the *R*^2^ obtained for hexanol (**A**) and 6-methyl-5-hepten-2-one (**B**).

The sensitivity (slopes of the calibration curves) for the different chemicals showed significant differences (*p* < 0.05) between the calibrations that either employed or did not employ an internal standard (IS) (Table S1). Figure 3 shows that, in the calibrations without the IS, the Y axis (left) represents the area (order of magnitude between 3 and 5), whereas, in the calibrations with the IS, the secondary Y axis (right) represents the ratio between the analyte standard area and the area of the IS (order of magnitude between −2 and −1). Thus, the difference between the order of magnitude makes it difficult to compare the different analytical curves and the sensitivity because it is related to the slope. However, when comparing the sensitivity of EC/AC and EC with IS/AC with IS, no significant differences (*p* < 0.05) were observed, although the AC calibrations showed higher standard deviations, similarly to what was observed for *RSD_b_*.

Therefore, due to the linear evaluation, the OLS methodology was selected, and the linear parameters and sensitivity shed light on a possibly better calibration methodology, EC being the one with lower errors and being more easily performed than AC or the calibrations with IS, which showed the least favorable results.

#### 3.2.3. Limit of Detection (LOD) and Limit of Quantification (LOQ)

**Figure 4:**
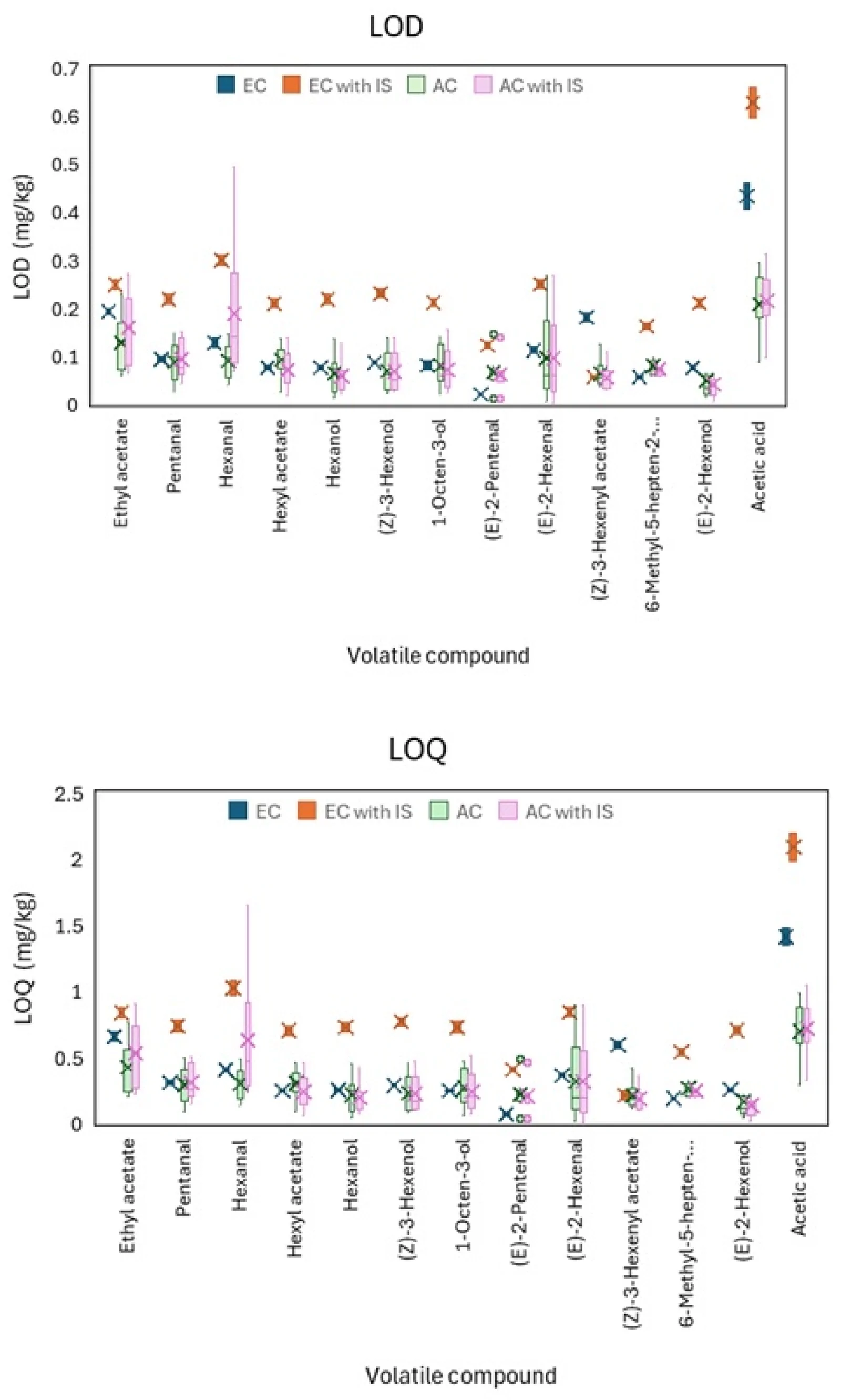
Box and whisker plots of LOD and LOQ for the four methodological calibrations (EC: blue, EC with IS: orange, AC: green, and AC with IS: sky blue) for each compound. The cross indicates the mean, the box is the 95% quartile, and the bars go from minimum to maximum if they are out of the box.

The limits of detection and quantification (LOD and LOQ, respectively) were determined, and both presented a similar behavior to that observed in *RSD_b_* (Table S1). Figure 4 shows the results obtained, and it can be seen that external standard calibration (EC) showed the least error and similar values to AC and AC with IS, the LOD ranging from 0.02 mg/kg ((*E*)-2-pentenal) to 0.43 mg/kg (acetic acid) and the LOQ ranging from 0.07 mg/kg to 1.42 mg/kg for the same compounds. The calibration that showed the highest LOD and LOQ values was the external standard with internal standard calibration (EC with IS), highlighting that the use of internal standards did not improve the methodology for quantifying volatiles in oily matrices. Furthermore, as for RSDb, the LOD and LOQ were also affected by the increase in the number of analyses for the calibration curves in the standard addition calibrations. Additionally, and following the previous trends observed, acetic acid showed the highest values of LOD and LOQ in all the calibrations. These results are in contrast with other values reported previously [27], but the difference could be due to the employment of a different preconcentration step (DHS against SPE).

As observed before for other parameters, the external matrix-matched standard calibration with an internal standard (EC with IS) showed the least favorable results, with the highest LOD and LOQ. Even though internal standards are recommended in chromatographic techniques, it seems to be detrimental to the quantification of virgin olive oil volatiles. This may be attributed to the fact that DHS tends to promote exhaustive extraction, a process highly dependent on the specific chemical properties of each analyte. Consequently, the use of a single internal standard (IS) to correct the response of all analytes may be suitable for certain compounds, but not universally applicable.

#### 3.2.4. Accuracy

Table 3 shows the relative recoveries obtained for the different volatile compounds with four calibrations at three different concentration levels. Among the different concentration levels (in the same calibration), no differences were detected, confirming similar behavior in the different concentrations. Additionally, EC and AC showed non-significant differences, with a maximum relative recovery of ±2%, except for the acetic acid. AC with IS showed an intermediate position, with poorer recovery results than EC or AC, but better than EC with IS, showing, in some cases, significant differences (*p* < 0.05). On the other hand, EC with IS showed the least favorable results, with a relative recovery ranging from 68.00 to 86.86%, except for acetic acid. Furthermore, EC showed the lowest standard deviations (SD); the deviation of the AC methodologies could be higher due to the high number of analyses.

The acetic acid exhibited the lowest recovery values among the volatile compounds analyzed. This behavior can be attributed to its strong hydrophilic nature, which makes its partitioning with the oily matrix of virgin olive oil difficult and consequently affects its extraction and quantification efficiency by DHS-GC-FID. In addition, the high volatility and low molecular weight of acetic acid cause losses during preconcentration, leading to recovery values ranging from 50.3% (with internal standard calibration) to 84.6% (with external calibration). Similar findings have been reported in interlaboratory studies, where acidic and polar volatiles showed a lower reproducibility compared to other compounds [12]. These results highlight the need for more robust analysis to improve the determination of acetic acid in virgin olive oil.

Thus, these results are in concordance with the results obtained before for linearity and sensitivity, highlighting the best performance of EC, with better results and lower errors associated, as well as the poor performance of the EC with IS methodology. AC and AC with IS showed optimal parameter values but had larger errors.

#### 3.2.5. Precision

The repeatability and intermediate precision values, expressed as the coefficient of variation (CV, %), are shown in Table 4. As expected, repeatability had lower CV values than intermediate precision due to the lower variability. However, no significant differences were observed between the different concentration levels, as was observed in accuracy.

Among the different calibrations, EC was the one that showed the lowest CV values, followed by EC with IS. EC repeatability ranged from 0.58% ((*E*)-2-pentenal) to 3.80% (hexyl acetate), except for acetic acid, which reached 9.89–10.81%; the intermediate precision ranged from 1.13% to 5.91%, except acetic acid, which ranged from 17.10% up to 18.78%. Standard addition calibrations showed higher values than the EC, which is explained by the increase in the number of analyses performed for these calibrations and the requirement of different matrices. AC showed CV values between 3.91% (repeatability of (*Z*)-3-hexenyl acetate) and 21.94% (intermediate precision of 6-methyl-5-hepten-2-one), except for the acetic acid. AC with IS showed CV values between the EC and AC, ranging from 2.42% (repeatability of 1-octen-3-ol) to 21.05% (intermediate precision of (*Z*)-3-hexenyl acetate), except for the acetic acid.

As mentioned before, and as for the other parameters, acetic acid was the volatile compound that showed the least favorable results, ranging from 9.89% (repeatability of EC) to 34.57% (intermediate precision of AC). These results may be due to the low lipophilicity of acetic acid making it difficult to solve in an oily matrix and having a random air/oil partitioning coefficient.

#### 3.2.6. Matrix Effect

As mentioned before, Figure 3 shows the four calibration curves (EC, EC with IS, AC, and AC with IS) for hexanol (Figure 3A) and 6-methyl-5-hepten-2-one (Figure 3B), considering only one (sample M5) of the nine curves obtained for AC and AC with IS calibrations. It can be observed that the slopes and interceptions seem to be parallel, which might be due to the possible absence of a matrix effect. The complexity of virgin olive oil, due to its rich and varied composition, might produce a matrix effect when determining some of its minor compounds.

The calibrations compared were EC against AC (the mean of the nine calibration curves obtained) and EC with IS against AC with IS (as for AC, it used the mean of the nine calibration curves obtained) for each compound; however, most of them should behave similarly. Calibrations with (EC with IS and AC with IS) and without (EC and AC) an internal standard are not comparable; even though significant differences between the slopes could be observed, they are due to the order of magnitude differences and not to matrix effects. The rest of the t tests employing the expanded uncertainty (Table S2) showed what was intuited in Figure 4: an absence of a matrix effect. For all the volatile compounds, including acetic acid, the calculated t was below U, statistically confirming the absence of a matrix effect.

After evaluating all the analytical parameters across all compounds, no consistent improvement attributable to the use of an IS was observed, suggesting that its inclusion does not enhance the overall analytical performance. While the use of deuterated analogs of each compound could potentially improve accuracy, their incorporation into a complex oily matrix such as virgin olive oil is impractical due to availability, cost, complexity, and lack of sense when working with FID instead of MS [35]. Furthermore, most of the compounds showed similar behavior, especially in the EC, except for acetic acid, being the one most difficult to quantify due to its lower precision.

Therefore, considering that AC and AC with IS were more time consuming and presented higher errors, these calibrations could be omitted for the quantification of volatile compounds in comparison with EC and EC with IS. After the evaluation of the linearity, LOD, LOQ, precision, and accuracy parameters, due to the low performance of the EC with IS calibration, it is preferable to avoid it, employing for the quantification the external matrix-matched calibration. Thus, EC with the application the OLS linear model adjustment seems to be the best statistical–analytical approach for quantifying volatiles in virgin olive oil.

### 3.3. Analytical–Statistical Method Application in VOO Volatile Quantification

Finally, the thirteen volatile compounds were quantified in VOO samples of different categories (three EVOOs, three VOOs, and three lampante olive oils), employing different calibration methods (Table S3). Although it was noted that the external matrix-matched calibration appeared to be the best and that no matrix effect was observed, it was of interest to assess whether there would be differences in the results obtained when analyzing real samples with the different methodological calibrations. In general, most compounds exhibited similar concentrations regardless of the calibration method employed. However, some exceptions were observed, such as ethyl acetate or 1-octen-3-ol, among others, which were quantified in certain samples by EC and/or EC with IC but not by the AC calibrations. This discrepancy is likely due to the differences in sensitivity and the lower LOD and LOQ values of the more sensitive methods, which permitted their quantification. In contrast, AC and AC with IS exhibited a lower sensitivity, preventing the quantification of these compounds in those samples. Additionally, the standard deviation (SD) was generally higher when using AC and AC with IS, whereas EC and EC with IS showed similar SD values across samples.

**Figure 5:**
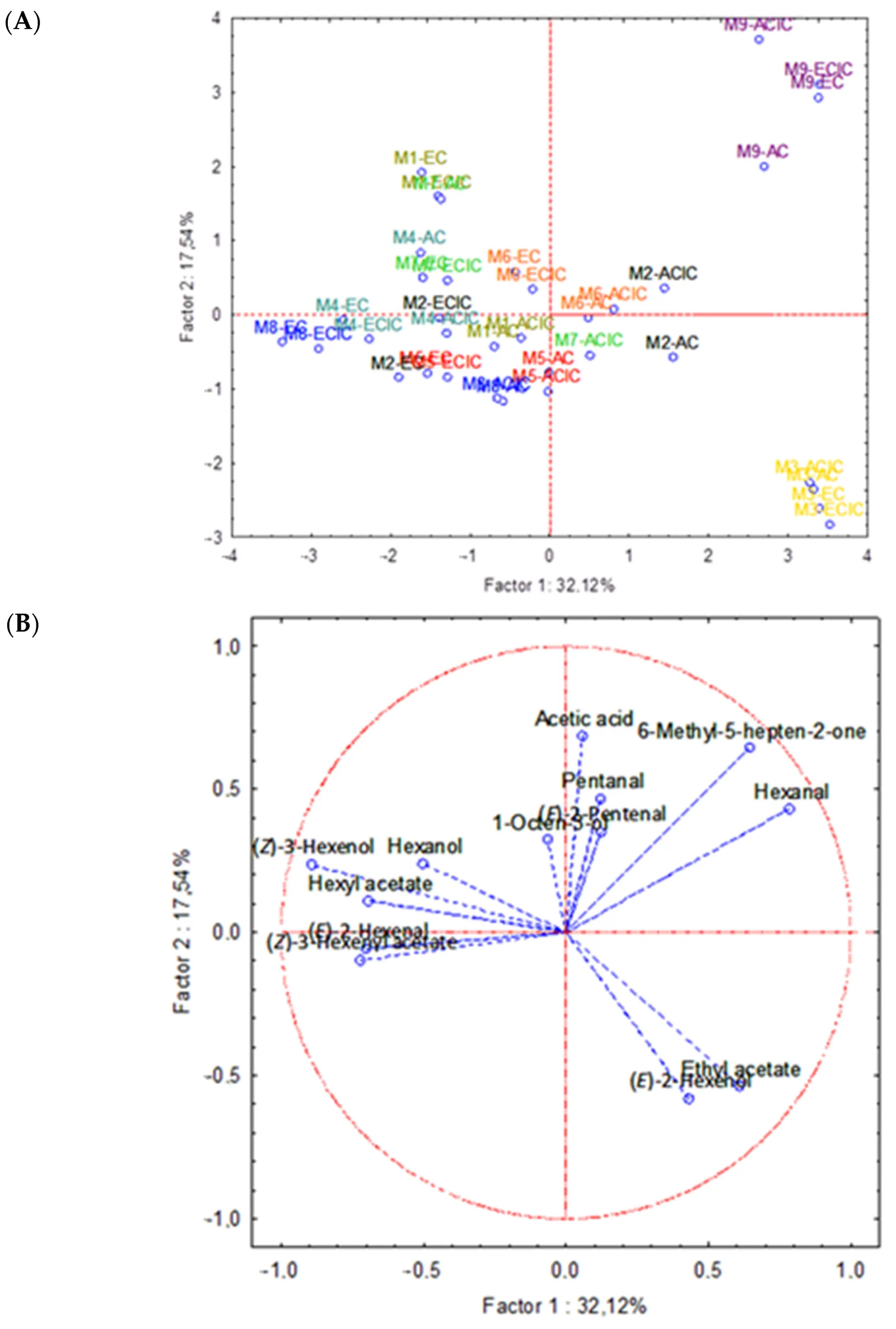
Loadings (**A**) and scores (**B**) of PCA for all the samples using different calibrations. In the loading, next to the sample code, appears the methodological calibration used.

A PCA (Figure 5) was applied to evaluate the effects of the category and the calibrations on the volatile compound quantifications of different virgin olive oils (EVOO, VOO, lampante). The PCA was performed using the thirteen volatile compounds as variables. From a sensory point of view, most of the thirteen compounds are green-related volatiles, except 1-octen-3-ol, 6-methyl-5-hepten-2-one, and acetic acid, which are related to sensory defects because they can be produced when non-sound olives are used to obtain the oil. Thus, Figure 5A plots the loadings; even though Factor 1 is 32.12%, any separation was observed among the samples, due to the non-employment of oxidation-related compounds. Equally, Factor 2 did not separate any sample or calibration. Factor 1 is mainly influenced by acetic acid, pentanal, and (*E*)-2-hexenol, whereas Factor 2 is influenced by hexanal, (*Z*)-3-hexenol, and hexyl acetate, among others. Even though samples or calibration methods cannot be clearly separated, certain samples showed slight differences between different calibrations, mainly EC/EC with IS with AC/AC with IS, possibly due to the wider error of this last calibration or to the differences observed in the method evaluation. Even the samples that belong to different categories are not well separated due to the selection of variables unrelated to oxidation or non-sound olives, as mentioned before. Additionally, it can be observed that the sum of the factors did not reach 50%; thus, no separation or trend can be observed. The lack of sample separation, even though they are different categories and varieties, can be explained by the volatiles chosen. Although the samples represent different varieties and categories, the selected volatile compounds were primarily focused on green/fruity attributes, with only a few related to sensory defects.

Figure 5B shows the scores and highlights that sample M9 (lampante) is strongly influenced by hexanal and 6-methyl-5-hepten-2-one, whereas sample M4 (EVOO) is separated due to ethyl acetate and (E)-2-hexenal. The rest of the samples are more or less influenced by the other volatiles, such as M8 (lampante), which is influenced by hexyl acetate or (*Z*)-3-hexenyl acetate.

All these results can be related to the sensory analyses, where sample M9 showed the highest intensity of defects (described as rancid by panelists) and the lowest mean of fruitiness. This could be due to the high content of hexanal (38.51–43.55 mg/kg), which is an oxidation-derived volatile compound that, in high concentrations, is associated with rancidity [28]. On the other hand, sample M3 showed the highest mean of fruitiness and, equally, showed a high concentration of ethyl acetate and (*E*)-2-hexenal, related to a green aroma.

Therefore, the methodological calibration did not influence the quantification of volatiles or, consequently, the separation of different virgin olive oil samples from different categories using the selected volatiles.

## 4. Conclusions

This study develops and validates an analytical–statistical approach for the quantification of volatile compounds in virgin olive oils by using different linear model adjustments, four calibrations, different parameters (accuracy, precision, and LOD, among others), and the evaluation of matrix effects. External matrix-matched calibration (EC) was established as the most precise and accurate strategy, showing the lowest limits of detection (LOD) and quantification (LOQ), the smallest relative standard deviations (*RSD_b_*), consistent results across different samples, and the best recovery rates. AC and AC with IS demonstrated a higher variability due to the diverse matrices and minor compound contents in different samples, requiring a separate validation for each analysis. EC with IS yielded the least favorable results, potentially due to the inadequate solubility of the internal standard in the lipid matrix, indicating the necessity for alternative standards. Visual analysis and statistical tests confirmed the absence of significant matrix effects among the compared calibration curves (EC vs. AC and EC with IS vs. AC with IS). This conclusion was supported by the calculated parameters *R*, $S_{R}^{2}$ , and *t*, which indicated no statistically significant differences between the slopes of the calibration curves.

## Figures and Tables

**Table 1:** Information and classification of the samples.

Labeled	M1	M2	M3	M4	M5	M6	M7	M8	M9
Variety	Picual	Arbequina	Coratina	Hojiblanca	Coratina	Mixture (not specified)	Picuda	Arbequina	Cornicabra
Harvesting	2022/23	2022/23	2022/23	2022/23	2022/23	2022/23	2016/17	2016/17	2016/17
Category	EVOO	EVOO	EVOO	VOO	VOO	VOO	Lampante	Lampante	Lampante
Origin	Andalusia (Spain)								
Sensory analysis									
Md ^a^	0	0	0	0.5	1.0	0.5	3.0	2.8	4.2
Mf ^b^	2.0	2.5	4.5	1.0	0.5	0.5	1.0	1.5	0.5
Physico-chemical parameters									
Acidity index ^c^	0.61	0.54	0.23	1.34	1.01	1.64	3.16	2.34	3.01
Peroxide value ^d^	8.22	7.63	12.57	14.13	10.94	9.86	22.61	23.85	19.21
K_232_	1.25	1.46	0.98	2.14	2.47	2.56	2.49	2.86	3.01
K_270_	0.09	0.14	0.19	0.21	0.17	0.25	0.18	0.22	0.26
ΔK	≤0.01	≤0.01	≤0.01	≤0.01	≤0.01	≤0.01	-	-	-

^a^ Median of sensory defects detected by the panelists. ^b^ Median of fruity sensory attribute detected by the panelists. ^c^ The acidity index is expressed as the grams of oleic acid per 100 g of virgin olive oil. ^d^ Peroxide value expressed as the milliequivalent (mEq) of O_2_ per kg.

**Table 2:** Results of the $F_{cal}$ , $t_{cal,a}$ , $t_{cal,b}$ , $t_{cal}$ , and $F_{Mandel}$ .

Volatile Compounds	$F_{cal}$ ^1^	$t_{cal,a}$ ^2^	$t_{cal,b}$ ^3^	$t_{cal}$ ^4^	$F_{Mandel}$ ^5^
Ethyl acetate	9.65	5.17	15.99	39.19	1.13
Pentanal	6.97	6.14	33.76	82.71	0.94
Hexanal	7.81	7.27	25.48	62.41	2.01
Hexyl acetate	2.53	5.29	41.38	101.35	1.67
Hexanol	3.26	4.11	41.64	101.99	0.68
(*Z*)-3-hexenol	7.27	2.75	35.53	87.07	1.21
1-Octen-3-ol	9.84	3.25	42.08	103.07	1.98
(*E*)-2-Pentenal	10.14	3.03	150.18	367.85	1.46
(*E*)-2-Hexenal	11.94	6.94	28.40	69.56	0.85
(*Z*)-3-Hexenyl acetate	2.87	4.08	17.51	42.88	1.12
6-Methyl-5-hepten-2-one	4.51	3.11	55.49	135.92	1.06
(*E*)-2-Hexenol	5.02	3.76	41.41	101.43	0.79
Acetic acid	11.05	2.18	7.40	18.14	2.11

Note: ^1^ *F_cal_* is the F of Fisher calculated following Equation (1). ^2^ *t_cal,a_* is the *t* student of the intercept, calculated following Equation (2); ^3^ *t_cal,b_* is the t student of the slope, calculated following Equation (3); ^4^ *t_cal_* is the *t* student calculated for the calibration curve following Equation (4); ^5^ *F_Mandel_* is the F test performed to determine the linear or quadratic fit of the calibration curve.

**Table 3:** Relative recovery at three concentration levels (0.1, 1, 10 mg/kg) for the four calibration methodologies.

Chemical Compound	Concentration Level (mg/kg)	EC Relative Recovery ± SD (%)	EC with IS Relative Recovery ± SD (%)	AC Relative Recovery ± SD (%)	AC with IS Relative Recovery ± SD (%)
Ethyl acetate	0.1	101.60 ±0.16 ^a^	70.48 ± 0.18 ^b^	101.28 ± 0.44 ^a^	95.02 ± 0.41 ^c^
	1	100.47 ± 0.18 ^a^	71.39 ± 0.15 ^b^	98.92 ± 0.60 ^a,c^	97.14 ± 0.52 ^c^
	10	98.55 ± 0.11 ^a,c^	74.06 ± 0.08 ^b^	101.96 ± 1.01 ^a^	96.05 ± 0.87 ^c^
(*Z*)-3-hexenyl acetate	0.1	100.18 ± 0.09 ^a^	86.86 ± 0.12 ^b^	98.36 ± 0.54 ^a^	100.01 ± 0.63 ^a^
	1	99.60 ± 0.10 ^a^	79.11 ± 0.27 ^b^	101.65 ± 0.98 ^a^	101.90 ± 0.98 ^a^
	10	101.02 ± 0.09 ^a,c^	83.57 ± 0.11 ^b^	98.37 ± 0.32 ^a^	101.91 ± 0.59 ^c^
1-octen-3-ol	0.1	101.04 ± 0.12 ^a^	71.77 ± 0.24 ^b^	100.29 ± 0.51 ^a^	102.23 ± 0.97 ^a^
	1	100.26 ± 0.13 ^a^	79.07 ± 0.22 ^b^	98.40 ± 0.81 ^a^	103.18 ± 0.37 ^c^
	10	98.94 ± 0.08 ^a^	75.2 ± 0.02 ^b^	98.56 ± 0.34 ^a^	101.97 ± 0.19 ^c^
(*E*)-2-pentenal	0.1	100.91 ± 0.03 ^a^	84.55 ± 0.15 ^b^	98.28 ± 1.03 ^a,c^	96.38 ± 0.86 ^c^
	1	101.78 ± 0.16 ^a^	80.88 ± 0.21 ^b^	100.53 ± 0.67 ^a^	95.83 ± 0.77 ^c^
	10	101.11 ± 0.12 ^a^	78.90 ± 0.04 ^b^	99.53 ± 0.76 ^a^	98.80 ± 0.45 ^a^
(*E*)-2-hexenol	0.1	99.33 ± 0.09 ^a^	68.62 ± 0.05 ^b^	101.23 ± 0.48 ^a^	104.67 ± 0.31 ^c^
	1	101.48 ± 0.08 ^a,c^	72.58 ± 0.11 ^b^	100.56 ± 0.52 ^a^	102.99 ± 0.40 ^c^
	10	99.05 ± 0.17 ^a^	68.89 ± 0.18 ^b^	100.53 ± 0.99 ^a^	104.50 ± 0.64 ^c^
6-methyl-5-hepten-2-one	0.1	98.37 ± 0.23 ^a^	68.65 ± 0.12 ^b^	99.38 ± 0.49 ^a^	96.97 ± 0.31 ^c^
	1	100.68 ± 0.31 ^a^	77.07 ± 0.15 ^b^	98.18 ± 0.88 ^a^	98.31 ± 0.51 ^a^
	10	99.92 ± 0.28 ^a^	75.98 ± 0.19 ^b^	98.79 ± 1.07 ^a,c^	96.69 ± 0.84 ^c^
Pentanal	0.1	98.91 ± 0.09 ^a^	77.32 ± 0.11 ^b^	101.14 ± 0.89 ^a^	101.93 ± 0.46 ^a^
	1	100.21 ± 0.24 ^a^	78.66 ± 0.10 ^b^	101.09 ± 0.76 ^a^	100.45 ± 0.72 ^a^
	10	99.16 ± 0.30 ^a^	76.00 ± 0.08 ^b^	98.12 ± 0.92 ^a^	100.56 ± 0.91 ^a^
Hexanal	0.1	98.59 ± 0.08 ^a^	74.48 ± 0.21 ^b^	100.31 ± 1.00 ^a^	101.03 ± 1.04 ^a^
	1	99.50 ± 0.14 ^a,c^	71.13 ± 0.16 ^b^	98.51 ± 0.84 ^a^	102.53 ± 0.32 ^c^
	10	99.47 ± 0.23 ^a^	74.06 ± 0.17 ^b^	98.95 ± 0.77 ^a^	104.12 ± 0.61 ^c^
Hexyl acetate	0.1	98.51 ± 0.08 ^a^	75.92 ± 0.22 ^b^	99.70 ± 0.43 ^a^	98.15 ± 0.53 ^a^
	1	98.02 ± 0.12 ^a^	77.38 ± 0.31 ^b^	100.58 ± 0.86 ^a^	101.43 ± 1.02 ^a^
	10	100.44 ± 0.31 ^a^	75.62 ± 0.28 ^b^	98.38 ± 0.74 ^a^	98.40 ± 0.79 ^a^
Hexan-1-ol	0.1	98.37 ± 0.07 ^a^	83.49 ± 0.14 ^b^	99.17 ± 0.91 ^a^	97.27 ± 0.64 ^a^
	1	99.35 ± 0.16 ^a^	84.12 ± 0.25 ^b^	100.80 ± 0.66 ^a^	97.80 ± 0.35 ^c^
	10	99.94 ± 0.31 ^a^	79.35 ± 0.16 ^b^	98.70 ± 0.81 ^a^	101.00 ± 0.76 ^a^
(*Z*)-3-hexenol	0.1	100.46 ± 0.19 ^a^	66.46 ± 0.23 ^b^	101.66 ± 0.63 ^a^	99.86 ± 0.92 ^a^
	1	101.83 ± 0.08 ^a^	68.00 ± 0.18 ^b^	100.62 ± 0.91 ^a^	101.48 ± 0.84 ^a^
	10	98.92 ± 0.17 ^a^	71.89 ± 0.27 ^b^	100.18 ± 0.84 ^a^	99.74 ± 0.62 ^a^
(*E*)-2-hexenal	0.1	98.11 ± 0.04 ^a^	73.40 ± 0.16 ^b^	99.61 ± 0.37 ^a^	103.50 ± 0.49 ^c^
	1	99.73 ± 0.18 ^a^	68.51 ± 0.31 ^b^	101.76 ± 0.96 ^a^	101.52 ± 1.00 ^a^
	10	101.58 ± 0.09 ^a^	72.53 ± 0.28 ^b^	99.21 ± 0.75 ^a^	102.46 ± 1.02 ^a^
Acetic acid	0.1	84.63 ± 0.84 ^a^	51.58 ± 1.01 ^b^	83.21 ± 1.49 ^a^	78.42 ± 1.06 ^c^
	1	82.87 ± 0.92 ^a^	56.34 ± 0.96 ^b^	86.71 ± 1.67 ^a^	76.99 ± 0.99 ^c^
	10	81.83 ± 0.87 ^a^	50.30 ± 0.97 ^b^	81.88 ± 1.52 ^a^	76.08 ± 1.14 ^c^

Note: Values with different letters (a, b, c) within the same row are significantly different (*p* < 0.05). Experiments were set up in triplicates.

**Table 4:** Repeatability and intermediate precision coefficients of variation (CV, %) at three concentration levels (0.1, 1, 10 mg/kg) for the four calibration methodologies.

Chemical Compound	Concentration Level (mg/kg)	EC		EC with IS		AC		AC with IS	
		Repeatability CV (%)	Intermediate Precision CV (%)	Repeatability CV (%)	Intermediate Precision CV (%)	Repeatability CV (%)	Intermediate Precision CV (%)	Repeatability CV (%)	**Intermediate Precision CV (%)**
Ethyl acetate	0.1	2.07	2.78	2.30	3.19	5.29	8.68	4.95	8.05
	1	2.28	3.24	1.89	2.58	7.13	12.05	6.21	10.36
	10	1.50	1.74	1.69	2.01	11.85	20.68	10.24	17.73
(*Z*)-3-hexenyl acetate	0.1	1.27	1.29	1.61	1.93	6.44	10.79	7.13	12.05
	1	1.38	1.53	3.34	5.10	11.50	20.05	12.50	21.05
	10	1.26	1.24	1.52	1.72	3.91	6.16	7.02	11.84
1-octen-3-ol	0.1	1.61	1.94	2.99	4.47	6.10	10.15	11.39	19.83
	1	1.73	2.16	2.75	4.05	9.55	16.43	4.49	7.21
	10	1.14	1.29	1.46	2.07	4.14	6.58	2.42	3.42
(*E*)-2-pentenal	0.1	0.58	1.13	1.98	2.56	12.08	21.10	10.12	17.52
	1	2.05	2.66	2.64	3.82	7.94	13.57	9.09	15.63
	10	1.60	1.95	1.70	2.31	8.96	15.15	5.41	8.89
(*E*)-2-hexenol	0.1	1.32	1.18	1.81	2.47	5.75	9.52	3.80	5.94
	1	1.17	1.46	1.50	1.75	6.21	10.36	4.83	7.84
	10	2.18	3.01	2.28	3.15	11.62	20.26	7.54	12.98
6-methyl-5-hepten-2-one	0.1	2.86	4.25	1.62	1.95	5.87	9.73	3.72	5.34
	1	3.79	5.91	1.96	2.55	10.35	17.94	6.10	10.15
	10	3.45	5.31	2.42	3.42	12.54	21.94	9.59	17.04
Pentanal	0.1	1.17	1.31	1.54	1.74	10.47	18.15	5.52	9.10
	1	2.99	4.47	1.38	1.53	8.97	15.42	8.51	14.57
	10	3.68	5.73	1.91	2.62	10.81	18.78	10.70	18.57
Hexanal	0.1	1.51	1.92	2.65	3.84	11.73	20.47	12.19	21.31
	1	1.84	2.37	2.09	2.77	9.82	17.10	3.91	6.16
	10	2.88	4.26	2.19	3.02	9.09	15.63	7.25	12.26
Hexyl acetate	0.1	1.43	1.84	2.76	4.01	5.18	8.47	6.33	10.57
	1	1.54	1.87	3.80	5.94	10.12	17.52	11.47	20.61
	10	3.80	5.82	3.45	5.31	8.74	14.99	9.32	16.05
Hexan-1-ol	0.1	1.01	1.89	1.84	2.37	10.71	18.54	7.59	12.89
	1	2.18	2.79	3.11	4.68	7.82	13.31	4.26	6.79
	10	3.67	5.83	2.02	2.71	9.54	16.47	8.97	15.42
(*Z*)-3-hexenol	0.1	2.42	3.42	2.88	4.26	7.48	12.68	10.81	18.78
	1	1.15	1.99	2.31	3.21	10.64	18.57	9.89	17.10
	10	2.19	3.00	3.31	5.12	9.89	17.09	7.36	12.47
(*E*)-2-hexenal	0.1	0.69	1.28	2.07	2.79	4.49	7.21	5.87	9.73
	1	2.30	3.21	3.81	5.93	11.27	19.62	11.73	20.47
	10	1.09	1.39	3.46	5.28	8.86	15.20	11.96	20.89
Acetic acid	0.1	9.89	17.10	11.85	20.68	17.37	30.78	12.42	21.73
	1	10.81	18.78	11.27	19.62	19.44	34.57	11.62	20.26
	10	10.24	17.73	11.39	19.83	17.71	31.41	13.34	23.41

## Data Availability

The original contributions presented in this study are included in the article/Supplementary Materials. Further inquiries can be directed to the corresponding authors.

## Supplementary Materials

The following supporting information can be downloaded at https://www.mdpi.com/article/10.3390/foods14193439/s1. Table S1 provides all the validation parameters for the different calibrations analyzed. Table S2 shows the results of *R*, $S_{R}^{2}$ , t , and *U* in the evaluation of the matrix effect through the comparison of the calibration curves used for each analyte. Lastly, Table S3 shows the concentrations of the volatile compounds in all the samples (M1–M10) quantified by the different calibrations. The loadings and scores of VOO and EVOO samples are shown in Figure S1.
