# Supplementary material for: Optimization of Calibration Strategies for the Quantification of Volatile Compounds in Virgin Olive Oil
Source: Foods. 2025 Oct 8;14(19):3439. doi: 10.3390/foods14193439 (PMC12524275; doi:10.3390/foods14193439)
Supplement: Supplementary file 1 [file foods-14-03439-s001.zip › foods-3893934-supplementary.pdf]

Supplementary material

Table S1. Summary of the precision parameters for the different calibrations.

External Standard Calibration (EC)

Compound	Calibration curve	Linearity		LOD (mg/kg)	LOQ (mg/kg)	Sensitivity
		**R^2**	**RSD_b (%)**			
Ethyl acetate	y=28921.0x+58921.0	0.9922	6.25	0.18	0.63	28921
Pentanal	y=22700.4x+4840.2	0.9983	2.96	0.09	0.30	22700
Hexanal	y=5766.3x+10369.3	0.9969	3.93	0.12	0.39	5766
Hexyl acetate	y=3702.8x-164.3	0.9988	2.41	0.07	0.24	3703
Hexanol	y=7910.2x+1323.1	0.9988	2.40	0.07	0.24	7910
(Z)-3-Hexenol	y=9950.5x+1317.6	0.9984	2.81	0.08	0.28	9951
1-Octen-3-ol	y=3002.9x+109.6	0.9989	2.38	0.07	0.24	3003
(E)-2-Pentenal	y=4773.1x+607.3	0.9999	0.67	0.02	0.07	4773
(E)-2-Hexenal	y=7898.2x+1647.4	0.9975	3.52	0.11	0.35	7898
(Z)-3-Hexenyl acetate	y=3116.1x-85.4	0.9935	5.71	0.17	0.57	3116
6-Methyl-5-hepten-2-one	y=4249.5x+1498.3	0.9994	1.80	0.05	0.18	4250
(E)-2-Hexenol	y=7423.7x+855.2	0.9988	2.41	0.07	0.24	7424
Acetic acid	y=6115.9x+11337.1	0.9648	13.51	0.41	1.35	6116

Note: None of the compounds showed outliers, and the residuals were in concordance with the three basic assumptions described.

External Standard with Internal Standard Calibration (EC with IS)

Compound	Calibration curve	Linearity		LOD (mg/kg)	LOQ (mg/kg)	Sensitivity
		**R^2**	**RSD_b (%)**			
Ethyl acetate	y=0.1521x+0.3273	0.9875	7.95	0.24	0.80	0.1521
Pentanal	y=0.1203x+0.0302	0.9905	6.93	0.21	0.69	0.1203
Hexanal	y=0.0304x+0.0575	0.9816	9.68	0.29	0.97	0.0304
Hexyl acetate	y=0.0197x-0.0004	0.9911	6.68	0.20	0.67	0.0197
Hexanol	y=0.0420x+0.0082	0.9905	6.93	0.21	0.69	0.0420
(Z)-3-Hexenol	y=0.0529x+0.0082	0.9892	7.39	0.22	0.74	0.0529
1-Octen-3-ol	y=0.0159x+0.0010	0.9908	6.81	0.20	0.68	0.0159

(E)-2-Pentenal	y=0.0253x+0.0037	0.9970	3.89	0.12	0.39	0.0253
(E)-2-Hexenal	y=0.0420x+0.0096	0.9872	8.04	0.24	0.80	0.0420
(Z)-3-Hexenyl acetate	y=0.0165x-0.0003	0.9994	1.78	0.05	0.18	0.0165
6-Methyl-5-hepten-2-one	y=0.0226x+0.0083	0.9947	5.15	0.16	0.52	0.0226
(E)-2-Hexenol	y=0.0395x+0.0052	0.9911	6.71	0.20	0.67	0.0395
Acetic acid	y=0.0323x+0.0634	0.9270	19.84	0.59	1.98	0.0323

Note: None of the compounds showed outliers, and the residuals were in concordance with the three basic assumptions described.

Standard Addition Calibration (AC)

Compound	Sample	Calibration curve	Linearity		LOD (mg/kg)	LOQ (mg/kg)	Sensitivity
			**R^2**	**RSDb (%)**			
Ethyl acetate	M1	y=51717.5x+65990.5	0.9992	2.04	0.06	0.20	51718
	M2	y=38412.1x+352642.0	0.9989	2.32	0.07	0.23	38412
	M3	y=61246.1x-7544.7	0.9962	4.36	0.13	0.44	61246
	M4	y=46465.7x+49250.5	0.9921	6.31	0.19	0.63	46466
	M5	y=64375.7x-6425.3	0.9884	7.66	0.23	0.77	64376
	M6	y=56742.4x+6926.6	0.9988	2.49	0.08	0.25	56742
	M7	y=38412.1x+352642.0	0.9989	2.33	0.07	0.23	38412
	M8	y=61994.5x+9277.9	0.9975	3.57	0.11	0.36	61995
	M9	y=33375.0x+62.7	0.9952	4.93	0.15	0.49	33375
	Mean	y=(51751.2±10741.4)x+(59747.6±113910.0)	0.9957±0.0036	4.28±1.90	0.13±0.06	0.42±0.19	51751±10741
Pentanal	M1	y=28060.9x+20568.7	0.9992	2.03	0.06	0.20	28061
	M2	y=28263.0x+23032.5	0.9979	3.25	0.10	0.33	28263
	M3	y=31645.3x+14645.6	0.9978	7.67	0.23	0.77	28520
	M4	y=28779.4x+18077.8	0.9998	0.88	0.03	0.09	28779
	M5	y=27137.6x+9184.8	0.9996	1.42	0.04	0.14	27138
	M6	y=31564.9x+186.1	0.9953	4.88	0.15	0.49	31565
	M7	y=20156.0x+12485.3	0.9988	2.49	0.08	0.25	20156
	M8	y=30204.7x+17394.9	0.9978	3.30	0.10	0.33	30205
	M9	y=25155.2x+16217.4	0.9952	4.93	0.15	0.49	25155
	Mean	y=(27885.2±3566.9)x+(14643.7±6808.1)	0.9979±0.0017	2.95±1.40	0.09±0.04	0.30±0.14	27885±3567
Hexanal	M1	y=6817.1x+23314.1	0.9997	1.31	0.04	0.13	6817

	M2	y=6757.1x+25915.0	0.9975	3.53	0.11	0.35	6757
	M3	y=7539.6x+12714.9	0.9953	4.85	0.15	0.49	7540
	M4	y=6704.0x+13539.4	0.9997	1.30	0.04	0.13	6704
	M5	y=5974.0x+9769.4	0.9987	2.56	0.08	0.26	5974
	M6	y=7688.7x+14382.1	0.9962	4.40	0.13	0.44	7689
	M7	y=5555.2x+84879.6	0.9988	2.44	0.07	0.24	5555
	M8	y=6859.1x+41810.7	0.9974	3.59	0.11	0.36	6859
	M9	y=9599.6x+214855.0	0.9979	3.26	0.10	0.33	9600
	Mean	y=(7054.9±1163.8)x+(49020.0±66474.7)	0.9979±0.0015	3.03±1.24	0.09±0.04	0.30±0.12	7055±1164
Hexyl acetate	M1	y=3613.1x+100.9	0.9999	0.86	0.03	0.09	3613
	M2	y=3490.8x-237.1	0.9969	3.96	0.12	0.40	3491
	M3	y=3364.9x+192.1	0.9986	2.65	0.08	0.27	3365
	M4	y=3411.7x+923.5	0.9958	4.59	0.14	0.46	3412
	M5	y=3610.1x-185.3	0.9990	2.23	0.07	0.22	3610
	M6	y=3967.8x-247.9	0.9973	3.68	0.11	0.37	3967
	M7	y=3485.4x-514.6	0.9982	3.03	0.09	0.30	3485
	M8	y=3539.3x+195.0	0.9982	2.99	0.09	0.30	3539
	M9	y=3617.8x-656.6	0.9973	3.68	0.11	0.37	3617
	Mean	y=(3566.8±175.2)x-(47.8±469.8)	0.9979±0.0012	3.07±1.10	0.09±0.03	0.31±0.11	3567±175
Hexanol	M1	y=7732.5x+4966.6	0.9998	1.00	0.30	0.10	7733
	M2	y=7259.1x+3906.0	0.9999	0.47	0.01	0.05	7259
	M3	y=8263.8x+3838.6	0.9993	1.88	0.06	0.19	8264
	M4	y=7350.8x+7147.7	0.9959	4.56	0.14	0.46	7351
	M5	y=7587.0x+1857.6	0.9999	0.78	0.02	0.08	7587
	M6	y=8242.9x+5609.7	0.9986	2.61	0.08	0.26	8243
	M7	y=7220.6x+3275.1	0.9987	2.57	0.08	0.26	7221
	M8	y=7497.3+4240.9	0.9987	2.51	0.08	0.25	7497
	M9	y=7428.2x+3679.9	0.9980	3.19	0.10	0.32	7428
	Mean	y=(7620.2±392.1)+(4280.2±1501.6)	0.9988±0.0013	2.17±1.30	0.07±0.04	0.22±0.13	7620±392
(Z)-3-Hexenol	M1	y=9679.4x+14745.8	0.9999	0.77	0.02	0.08	9679
	M2	y=9651.7x+7104.4	0.9998	0.89	0.03	0.09	9652
	M3	y=10262.1x+18475.7	0.9982	2.98	0.09	0.30	10262
	M4	y=9204.5x+14111.1	0.9958	4.60	0.14	0.46	9205
	M5	y=9444.7x+11203.7	0.9998	1.13	0.03	0.11	9445
	M6	y=10367.1x+15046.8	0.9991	2.07	0.06	0.21	10367

	M7	y=9105.6x+1687.9	0.9994	1.72	0.05	0.17	9106
	M8	y=9439.2x+9997.0	0.9988	2.48	0.07	0.25	9439
	M9	y=9163.2x+6467.8	0.9967	4.10	0.12	0.41	9163
	Mean	y=(9590.8±457.8)x+(10982.3±5237.5)	0.9986±0.0015	2.30±1.37	0.07±0.04	0.23±0.14	9591±458
1-Octen-3-ol	M1	y=2872.2x+35.6	0.9998	1.12	0.03	0.11	2872
	M2	y=2908.0x-629.9	0.9964	4.26	0.13	0.43	2908
	M3	y=3104.1x-221.1	0.9991	2.07	0.06	0.21	3104
	M4	y=2767.1x+284.5	0.9955	4.74	0.14	0.47	2767
	M5	y=2842.0x-179.0	0.9999	0.67	0.02	0.07	2842
	M6	y=3259.4x-633.6	0.9968	4.03	0.12	0.40	3259
	M7	y=2806.1x-202.1	0.9986	2.70	0.08	0.27	2806
	M8	y=2794.0x+9.5	0.9990	2.24	0.07	0.22	2794
	M9	y=2918.7x-216.8	0.9988	2.46	0.07	0.25	2919
	Mean	y=(2919.1±162.0)x-(194.8±297.5)	0.9982±0.0016	2.70±1.40	0.08±0.04	0.27±0.14	2919±162
(E)-2-Pentenal	M1	y=7548.5x-907.1	0.9953	4.89	0.15	0.49	7549
	M2	y=6431.7x+1892.8	0.9990	2.20	0.07	0.22	6432
	M3	y=7332.0x+1837.6	0.9999	0.39	0.01	0.04	7332
	M4	y=6968.8x+959.9	0.9990	2.22	0.07	0.22	6969
	M5	y=7204.6x+877.7	0.9990	2.13	0.06	0.21	7205
	M6	y=7298.7x+8.4	0.9990	2.30	0.07	0.23	7299
	M7	y=5879.8x-153.3	0.9993	1.82	0.06	0.18	5880
	M8	y=7183.8x1418.5	0.9995	1.62	0.05	0.16	7183
	M9	y=5769.5x+1200.8	0.9986	2.63	0.08	0.26	5770
	Mean	y=(6846.4±657.3)x+(792.8±954.9)	0.9988±0.0014	2.25±1.18	0.07±0.04	0.23±0.12	6846±657
(E)-2-Hexenal	M1	y=9015.6x+20103.3	0.9990	2.21	0.07	0.22	9016
	M2	y=12916.8x+4236.2	0.9992	2.00	0.06	0.20	12917
	M3	y=7332.0x+1837.6	0.9999	0.39	0.01	0.04	7332
	M4	y=9975.5x+45054.1	0.9994	1.72	0.05	0.17	9976
	M5	y=9084.2x+5596.5	0.9997	1.14	0.03	0.11	9084
	M6	y=7298.7x+8.4	0.9990	2.30	0.07	0.23	7299
	M7	y=8134.2x+6125.9	0.9999	0.20	0.01	0.02	8134
	M8	y=9211.9x+3834.2	0.9998	1.08	0.03	0.11	9212
	M9	y=8613.1x+8629.3	0.9954	4.82	0.15	0.48	8613
	Mean	y=(9841.6±1477.8)+(19933.2±20337.4)	0.9964±0.0055	3.21±2.98	0.10±0.09	0.32±0.30	9842±1478
(Z)-3-Hexenyl acetate	M1	y=3310.4x+5241.1	0.9996	1.50	0.05	0.15	3310

	M2	y=3747.2x+4078.1	0.9994	1.62	0.05	0.16	3747
	M3	y=3560.5x+2656.6	0.9993	1.86	0.06	0.19	3561
	M4	y=3417.2x+4783.9	0.9986	2.61	0.08	0.26	3417
	M5	y=3348.1x+7028.6	0.9997	1.20	0.04	0.12	3348
	M6	y=3454.4x+3092.6	0.9985	2.74	0.08	0.27	3454
	M7	y=3176.0x-106.1	0.9997	1.24	0.04	0.12	3176
	M8	y=3412.2x+2807.3	0.0991	2.08	0.06	0.21	3412
	M9	y=3348.8x-499.2	0.9965	4.18	0.13	0.42	3349
	Mean	y=(3419.4±161.9)x+(3231.4±2428.2)	0.9989±0.0010	2.12±0.95	0.06±0.03	0.21±0.10	3419±162
6-Methyl-5-hepten-2-one	M1	y=4687.4x-182.4	0.9956	4.71	0.14	0.47	4687
	M2	y=4550.3x+2547.7	0.9992	2.04	0.06	0.20	4550
	M3	y=4836.8x-166.3	0.9987	2.51	0.08	0.25	4837
	M4	y=4691.0x+161.6	0.9984	2.83	0.09	0.28	4691
	M5	y=4674.3x-444.5	0.9992	2.02	0.06	0.20	4674
	M6	y=4727.7x+73.8	0.9989	2.31	0.07	0.23	4728
	M7	y=4397.8x+1509.8	0.9992	2.04	0.06	0.20	4398
	M8	y=4744.7x+1296.5	0.9988	2.41	0.07	0.24	4745
	M9	y=4481.0x+9769.8	0.9979	3.22	0.10	0.32	4481
	Mean	y=(4643.4±139.3)x+(1618.5±3213.6)	0.9984±0.0012	2.68±0.86	0.08±0.03	0.27±0.09	4643±139
(E)-2-Hexenol	M1	y=7702.0x+1769.1	0.9998	1.11	0.03	0.11	7702
	M2	y=7907.4x+11094.9	0.9999	0.71	0.02	0.07	7907
	M3	y=8100.7x+551.8	0.9997	1.19	0.04	0.12	8101
	M4	y=8040.1x+5432	0.9991	2.15	0.06	0.22	8040
	M5	y=7873.8x-46.9	0.9999	0.76	0.02	0.08	7874
	M6	y=8079.9x+3190.6	0.9993	1.89	0.06	0.19	8080
	M7	y=7245.3x+11891.4	0.9999	0.48	0.01	0.05	7245
	M8	y=7940.0x-19.3	0.9993	1.83	0.06	0.18	7940
	M9	y=7625.2x+40.6	0.9955	4.78	0.14	0.48	7625
	Mean	y=(7834.9±273.8)x+(3767.2±4742.2)	0.9992±0.0014	1.66±1.31	0.05±0.04	0.17±0.13	7835±274
Acetic acid	M1	y=13503.1x+82079.4	0.9810	9.83	0.30	0.98	13503
	M2	y=10765.2x+85978.7	0.9908	6.81	0.20	0.68	10766
	M3	y=9691.4x+13000.3	0.9873	8.03	0.24	0.80	9691
	M4	y=10800.0x+33283.4	0.9941	5.46	0.16	0.55	10800
	M5	y=10324.4x+15041.0	0.9823	9.50	0.29	0.95	10324
	M6	y=10347.3x+19389.6	0.9913	6.62	0.20	0.66	10347

M7	y=7431.5x+7087.8	0.9907	6.84	0.21	0.68	7432
M8	y=12318.4x+32277.6	0.9983	2.92	0.09	0.29	12318
M9	y=7108.2x+78181.1	0.9912	6.65	0.20	0.67	7108
Mean	y=(10254.4±2048.5)x+(40702.1±32215.6)	0.9897±0.0054	6.96±2.08	0.21±0.06	0.70±0.21	10254±2049

Note: None of the compounds showed outliers, and the residuals were in concordance with the three basic assumptions. It needs to be considered that for standard addition; a calibration curve is done for each sample in triplicate.

Standard Addition with Internal Standard Calibration (AC with IS)

Compound	Sample	Calibration curve	Linearity		LOD (mg/kg)	LOQ (mg/kg)	Sensitivity
			**R^2**	**RSD_b (%)**			
Ethyl acetate	M1	y=0.2618x+0.3136	0.9989	2.37	0.07	0.24	0.2618
	M2	y=0.2495x+0.3649	0.9908	6.82	0.21	0.68	0.2495
	M3	y=0.3123x-0.0560	0.9943	5.35	0.16	0.54	0.3123
	M4	y=0.2343x+0.2316	0.9982	3.03	0.09	0.30	0.2343
	M5	y=0.3265x-0.0379	0.9878	7.85	0.24	0.75	0.3265
	M6	y=0.2845x+0.0419	0.9990	2.20	0.07	0.22	0.2845
	M7	y=0.1956x+1.7278	0.9839	9.05	0.27	0.91	0.1956
	M8	y=0.3086x+0.0282	0.9946	5.21	0.16	0.52	0.3086
	M9	y=0.1598x+0.0252	0.9929	5.96	0.18	0.60	0.1598
	Mean	y=(0.2592±0.0560)x+(0.2933±0.5595)	0.9934±0.0052	5.32±2.42	0.16±0.07	0.53±0.24	0.2592±0.0560
Pentanal	M1	y=0.1417x+0.0964	0.9988	2.45	0.07	0.25	0.1417
	M2	y=0.1373x+0.1288	0.9954	4.79	0.14	0.48	0.1373
	M3	y=0.1614x+0.0625	0.9980	3.19	0.10	0.32	0.1614
	M4	y=0.1454x+0.0810	0.9987	2.59	0.08	0.26	0.1454
	M5	y=0.1377x+0.0436	0.9996	1.43	0.04	0.14	0.1377
	M6	y=0.1582x+0.0047	0.9960	4.47	0.13	0.45	0.1582
	M7	y=0.1005x+0.0618	0.9989	2.30	0.07	0.23	0.1005
	M8	y=0.1505x+0.0764	0.9950	5.02	0.15	0.50	0.1505
	M9	y=0.1198x+0.1031	0.9993	1.83	0.06	0.18	0.1198
	Mean	y=(0.1392±0.0191)x+(0.0732±0.0361)	0.9978±0.0018	3.12±1.33	0.09±0.04	0.31±0.13	0.1392±0.0191
Hexanal	M1	y=0.0348x+0.1122	0.9990	2.29	0.07	0.23	0.0348
	M2	y=0.0325x+0.1347	0.9918	6.43	0.19	0.64	0.0325
	M3	y=0.0386x+0.0601	0.9955	4.74	0.14	0.47	0.0386

	M4	y=0.0338x+0.0660	0.9980	3.16	0.10	0.32	0.0338
	M5	y=0.0304x+0.0480	0.9987	2.54	0.08	0.25	0.0304
	M6	y=0.0385x+0.0735	0.9972	3.75	0.11	0.38	0.0385
	M7	y=0.0287x+0.4157	0.9728	11.82	0.36	1.18	0.0287
	M8	y=0.0344x+0.2017	0.9940	5.48	0.16	0.55	0.0344
	M9	y=0.0414x+1.1071	0.9486	16.46	0.49	1.65	0.0414
	Mean	y=(0.0348±0.0041)x+(0.2466±0.3425)	0.9884±0.0170	6.30±4.79	0.19±0.14	0.63±0.48	0.0348±0.0041
Hexyl acetate	M1	y=0.0182x+0.0001	0.9995	1.55	0.05	0.16	0.0182
	M2	y=0.0170x+0.0003	0.9993	1.89	0.06	0.19	0.0170
	M3	y=0.0172x-0.0001	0.9974	3.65	0.11	0.37	0.0172
	M4	y=0.0172+0.0034	0.9996	1.37	0.04	0.14	0.0172
	M5	y=0.0183x-0.0011	0.9990	2.29	0.07	0.23	0.0183
	M6	y=0.0199x-0.0008	0.9979	3.25	0.10	0.33	0.0199
	M7	y=0.0174x-0.0024	0.9989	2.39	0.07	0.24	0.0174
	M8	y=0.0176x	0.9958	4.62	0.14	0.46	0.0176
	M9	y=0.0173x-0.0005	0.9999	0.62	0.02	0.06	0.0173
	Mean	y=(0.0178±0.0009)x-(0.0001±0.0016)	0.9986±0.0013	2.40±1.25	0.07±0.04	0.24±0.13	0.0178±0.0009
Hexanol	M1	y=0.0390x+0.0232	0.9994	1.81	0.05	0.18	0.0390
	M2	y=0.0353x+0.0229	0.9988	2.44	0.07	0.24	0.0353
	M3	y=0.0422x+0.0163	0.9992	2.02	0.06	0.20	0.0422
	M4	y= 0.0371x+0.0334	0.9997	1.16	0.04	0.12	0.0371
	M5	y=0.0385x+0.0088	0.9999	0.69	0.02	0.07	0.0385
	M6	y=0.0413x+0.0293	0.9991	2.11	0.06	0.21	0.0413
	M7	y=0.0360x+0.0163	0.9990	2.28	0.07	0.23	0.0360
	M8	y=0.0373x+0.0186	0.9964	4.27	0.13	0.43	0.0373
	M9	y=0.0354x+0.0248	0.9998	0.90	0.03	0.09	0.0354
	Mean	y=(0.0380±0.0025)x+(0.0215±0.0074)	0.9990±0.0011	1.96±1.07	0.06±0.03	0.20±0.11	0.0380±0.0025
(Z)-3-Hexenol	M1	y=0.0490x+0.0704	0.9994	1.69	0.05	0.17	0.0490
	M2	y=0.0469x+0.0401	0.9988	2.49	0.08	0.25	0.0469
	M3	y=0.0526x+0.0875	0.9984	2.80	0.08	0.28	0.0526
	M4	y=0.0464x+0.0679	0.9998	0.95	0.03	0.10	0.0464
	M5	y=0.0480x+0.0549	0.9998	1.11	0.03	0.11	0.0480
	M6	y=0.0520x+0.0773	0.9995	1.57	0.05	0.16	0.0520
	M7	y=0.0454x+0.0085	0.9998	0.96	0.03	0.10	0.0454
	M8	y=0.0470x+0.0461	0.9963	4.34	0.13	0.43	0.0470

	M9	y=0.0436x+0.0408	0.9957	4.67	0.14	0.47	0.00436
	Mean	y=(0.0479±0.0029)x+(0.0548±0.0240)	0.9986±0.0016	2.28±1.41	0.07±0.04	0.23±0.14	0.0479±0.0029
1-Octen-3-ol	M1	y=0.01446x-0.0001	0.9993	1.84	0.06	0.18	0.0145
	M2	y=0.0142x-0.0020	0.9947	5.14	0.15	0.51	0.0142
	M3	y=0.0158x-0.0020	0.9991	2.08	0.06	0.21	0.0158
	M4	y=0.0140x+0.0004	0.9996	1.42	0.04	0.14	0.0140
	M5	y=0.0144x-0.0010	0.9999	0.76	0.02	0.08	0.0144
	M6	y=0.0164x-0.0028	0.9974	3.62	0.11	0.36	0.0164
	M7	y=0.0140x-0.0009	0.9992	2.05	0.06	0.21	0.0140
	M8	y=0.0139x-0.0007	0.9970	3.87	0.12	0.39	0.0139
	M9	y=0.0140x+0.0012	0.9999	0.83	0.03	0.08	0.0140
	Mean	y=(0.0146±0.0009)x-(0.0009±0.0013)	0.9985±0.0017	2.40±1.49	0.07±0.05	0.24±0.15	0.0146±0.0009
(E)-2-Pentenal	M1	y=0.0366x-0.0041	0.9957	4.63	0.14	0.46	0.0366
	M2	y=0.0315x+0.0092	0.9990	2.21	0.07	0.22	0.0315
	M3	y=0.0360x+0.0090	0.9999	0.38	0.01	0.04	0.0360
	M4	y=0.0343x+0.0035	0.9995	1.53	0.06	0.15	0.0343
	M5	y=0.0351x+0.0045	0.9993	1.90	0.06	0.19	0.0351
	M6	y=0.0357x+0.0003	0.9990	2.26	0.07	0.23	0.0360
	M7	y=0.0286x-0.0008	0.9993	1.93	0.06	0.19	0.0286
	M8	y=0.0348x+0.0075	0.9995	1.59	0.05	0.16	0.0348
	M9	y=0.0280x+0.0066	0.9989	2.33	0.07	0.23	0.0280
	Mean	y=(0.0334±0.0032)x+(0.0040±0.0046)	0.9989±0.0012	2.08±1.12	0.06±0.03	0.21±0.11	0.0334±0.0032
(E)-2-Hexenal	M1	y=0.0435x+0.0989	0.9992	1.97	0.06	0.20	0.0435
	M2	y=0.0632x+0.0205	0.9992	2.00	0.06	0.20	0.0632
	M3	y=0.0524x+0.1255	0.9908	6.82	0.21	0.68	0.0524
	M4	y=0.0493x+0.2169	0.9982	2.98	0.09	0.30	0.0493
	M5	y=0.0443x+0.0275	0.9998	0.92	0.03	0.09	0.0443
	M6	y=0.0536x+0.2942	0.9843	8.95	0.67	0.90	0.0536
	M7	y=0.0396x+0.0299	0.9999	0.21	0.01	0.02	0.0396
	M8	y=0.0446x+0.0195	0.9999	0.72	0.02	0.07	0.0446
	M9	y=0.0418x+0.0435	0.9964	4.22	0.13	0.42	0.0418
	Mean	y=(0.0480±0.0074)x+(0.0974±0.0988)	0.9964±0.0054	3.19±2.98	0.10±0.09	0.32±0.30	0.0480±0.0074
(Z)-3-Hexenyl acetate	M1	y=0.0160x+0.0258	0.9997	1.20	0.04	0.12	0.0160
	M2	y=0.0183x+0.0200	0.9994	1.67	0.05	0.17	0.0183
	M3	y=0.0175x+0.0130	0.9993	1.90	0.06	0.19	0.0175

	M4	y=0.0168x+0.0226	0.9993	1.92	0.06	0.19	0.0168
	M5	y=0.0163x+0.0344	0.9998	1.00	0.03	0.10	0.0163
	M6	y=0.0169x+0.0152	0.9985	2.75	0.08	0.28	0.0169
	M7	y=0.0155x-0.0005	0.9998	1.02	0.03	0.10	0.0155
	M8	y=0.0165x+0.0140	0.9994	1.68	0.05	0.17	0.0165
	M9	y=0.0163x-0.0021	0.9974	3.62	0.11	0.36	0.0163
	Mean	y=(0.0167±0.0008)x+(0.0158±0.0118)	0.9992±0.0001	1.86±0.86	0.06±0.03	0.19±0.09	0.0167±0.0008
6-Methyl-5-hepten-2-one	M1	y=0.0227x-0.0007	0.9961	4.45	0.13	0.45	0.0227
	M2	y=0.0222x+0.0124	0.9991	2.07	0.06	0.21	0.0222
	M3	y=0.0237x-0.0009	0.9987	2.55	0.08	0.26	0.0237
	M4	y=0.0231x+0.0001	0.9990	2.20	0.07	0.22	0.0231
	M5	y=0.0228x-0.0021	0.9992	1.96	0.06	0.20	0.0228
	M6	y=0.0231x+0.0005	0.9989	2.32	0.07	0.23	0.0231
	M7	y=0.0214x+0.0074	0.9990	2.28	0.07	0.23	0.0214
	M8	y=0.0230x+0.0067	0.9992	2.02	0.06	0.20	0.0230
	M9	y=0.0217x+0.0484	0.9988	2.47	0.07	0.25	0.0218
	Mean	y=(0.0226±0.0007)x+(0.0080±0.0159)	0.9987±0.0010	2.48±0.77	0.07±0.02	0.25±0.08	0.0226±0.0007
(E)-2-Hexenol	M1	y=0.0373x+0.0090	0.9999	0.83	0.03	0.08	0.0373
	M2	y=0.0386x+0.0544	0.9999	0.70	0.02	0.07	0.0386
	M3	y=0.0397x+0.0027	0.9997	1.23	0.04	0.12	0.0397
	M4	y=0.0396x+0.0250	0.9997	1.31	0.04	0.13	0.0396
	M5	y=0.0384x-0.0001	0.9999	0.56	0.02	0.06	0.0384
	M6	y=0.0395x+0.0159	0.9993	1.94	0.06	0.19	0.0395
	M7	y=0.0352x+0.0582	0.9999	0.15	0.01	0.02	0.0352
	M8	y=0.0385x+0.0005	0.9996	1.45	0.04	0.15	0.0385
	M9	y=0.0370x+0.0011	0.9964	4.23	0.13	0.42	0.0370
	Mean	y=(0.0382±0.0015)x+(0.0185±0.0230)	0.9994±0.0011	1.38±1.20	0.04±0.04	0.14±0.12	0.0382±0.0015
Acetic acid	M1	y=0.0649x+0.4031	0.9786	10.45	0.31	1.05	0.0649
	M2	y=0.0523x+0.4227	0.9881	7.75	0.23	0.78	0.0523
	M3	y=0.0475x+0.0635	0.9871	8.10	0.24	0.81	0.0475
	M4	y=0.0533x+0.1597	0.9932	5.84	0.18	0.58	0.0533
	M5	y=0.0503x+0.0735	0.9833	9.21	0.27	0.92	0.0503
	M6	y=0.0506x+0.0952	0.9910	6.73	0.20	0.67	0.0506
	M7	y=0.0362x+0.0347	0.9904	6.95	0.21	0.70	0.0362
	M8	y=0.0595x+0.1590	0.9979	3.27	0.10	0.33	0.0595

M9	y=0.0343x+0.3838	0.9917	6.45	0.19	0.65	0.0343
Mean	y=(0.0499±0.0098)x+(0.1995±0.1585)	0.9891±0.0056	7.19±2.06	0.22±0.06	0.72±0.21	0.0499±0.0098

Note: None of the compounds showed outliers, and the residuals were in concordance with the three basic assumptions. It needs to be considered that for standard addition; a calibration curve is done for each sample in triplicate.

Table S2. Results of R, S_R^2, t and U in the evaluation of the matrix effect through the comparison of the calibration curves used for each analyte.

Compound	Compared calibrations	R	S_R^2	t	U	Matrix effect
Ethyl acetate	EC and AC	1.79	7.40	0.29	2	No
	EC with IS and AC with IS	1.70	7.58	0.26	2	No
Pentanal	EC and AC	1.23	1.57	0.18	2	No
	EC with IS and AC with IS	1.16	1.42	0.13	2	No
Hexanal	EC and AC	1.23	4.76	0.10	2	No
	EC with IS and AC with IS	1.14	4.89	0.06	2	No
Hexyl acetate	EC and AC	1.04	1.08	0.04	2	No
	EC with IS and AC with IS	1.11	1.23	0.10	2	No
Hexanol	EC and AC	1.04	1.11	0.04	2	No
	EC with IS and AC with IS	1.10	1.26	0.09	2	No
(*Z*)-3-Hexenol	EC and AC	1.04	1.10	0.04	2	No
	EC with IS and AC with IS	1.10	1.25	0.09	2	No
1-Octen-3-ol	EC and AC	1.03	1.06	0.03	2	No
	EC with IS and AC with IS	1.10	1.21	0.09	2	No
(*E*)-2-Pentenal	EC and AC	1.43	2.08	0.30	2	No
	EC with IS and AC with IS	1.32	1.77	0.24	2	No
(*E*)-2-Hexenal	EC and AC	1.25	1.62	0.19	2	No
	EC with IS and AC with IS	1.14	1.38	0.12	2	No
(*Z*)-3-Hexenyl acetate	EC and AC	1.10	1.21	0.09	2	No
	EC with IS and AC with IS	1.01	1.02	0.01	2	No
6-Methyl-5-hepten-2-one	EC and AC	1.09	1.32	0.08	2	No
	EC with IS and AC with IS	1.00	1.14	0.01	2	No
(*E*)-2-Hexenol	EC and AC	1.06	1.13	0.05	2	No
	EC with IS and AC with IS	1.00	1.09	0.03	2	No
Acetic acid	EC and AC	1.68	6.29	0.27	2	No

	EC with IS and AC with IS	1.54	6.26	0.22	2	No

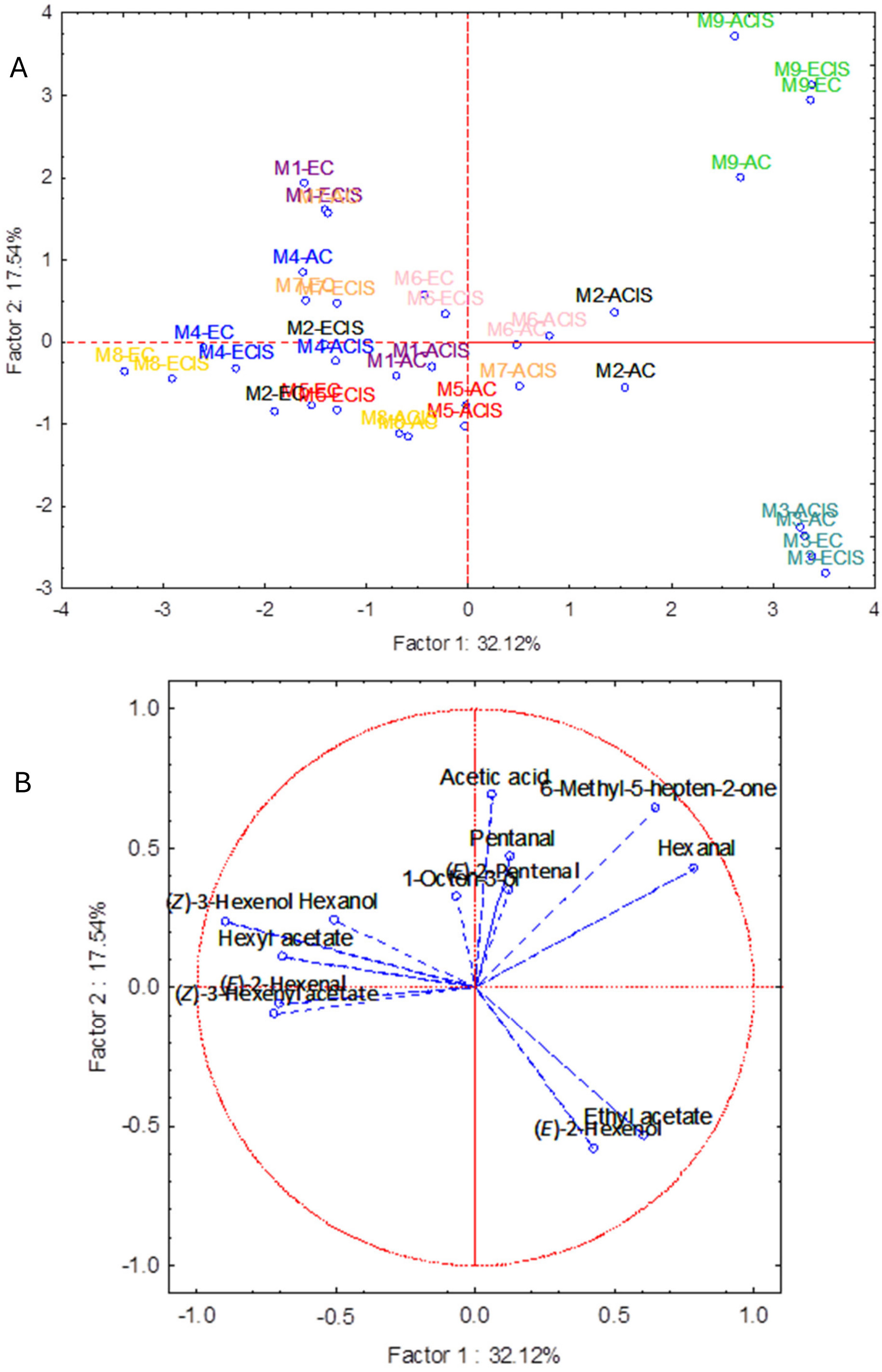

Figure S1. Loadings (A) and Scores (B) of a PCA of the EVOO, VOO and lampante samples quantifying the volatiles by the different methodological calibration. In the loading, next to the sample code appears the methodological calibration used.

Table S3. Concentration (mg/kg ± SD) of the volatile compounds in all the samples (M1-M10) quantified by the different calibrations.

Compound	Sample	Concentration by EC ± SD (mg/Kg)	Concentration by EC with IS ± SD (mg/Kg)	Concentration by AC ± SD (mg/Kg)	Concentration by AC with IS ± SD (mg/Kg)
Ethyl acetate	M1	1.88±0.06	1.77±0.05	N.d.	N.d.
	M2	1.46±0.11	1.31±0.13	N.d.	N.d.
	M3	0.22±0.12	N.d.	N.d.	N.d.
	M4	0.09±0.01	0.06±0.01	0.09±0.03	0.09±0.03
	M5	N.d.	N.d.	N.d.	N.d.
	M6	0.15±0.08	0.12±0.08	N.d.	N.d.
	M7	1.15±0.09	1.06±0.09	0.92±0.10	0.88±0.18
	M8	0.15±0.07	0.09±0.03	N.d.	N.d.
	M9	2.02±0.37	0.92±0.37	N.d.	0.06±0.02
Pentanal	M1	1.12±0.04	0.97±0.02	0.73±0.06	0.68±0.07
	M2	0.94±0.02	0.82±0.01	0.27±0.02	0.29±0.03
	M3	1.00±0.01	0.87±0.01	0.34±0.03	0.39±0.03
	M4	0.36±0.02	0.34±0.02	0.22±0.02	0.27±0.04
	M5	0.31±0.02	0.21±0.02	0.14±0.10	0.12±0.10
	M6	0.25±0.04	0.18±0.03	0.11±0.02	0.11±0.03
	M7	0.62±0.01	0.62±0.01	0.51±0.01	0.52±0.02
	M8	0.93±0.08	0.80±0.07	0.58±0.02	0.51±0.02
	M9	0.86±0.05	0.71±0.04	0.65±0.07	0.60±0.10
Hexanal	M1	1.69±0.08	1.42±0.10	1.18±0.11	1.22±0.13
	M2	1.18±0.09	0.97±0.07	0.74±0.14	0.85±0.14
	M3	0.54±0.02	0.28±0.02	0.24±0.06	0.26±0.11
	M4	2.02±0.11	1.95±0.10	0.85±0.27	0.56±0.25
	M5	2.50±0.10	2.45±0.10	1.64±0.14	1.59±0.28
	M6	2.07±0.18	1.92±0.16	1.59±0.42	1.34±0.39
	M7	18.41±0.24	16.78±0.22	15.28±0.30	15.48±0.34
	M8	8.22±0.14	7.37±0.17	6.10±0.26	5.88±0.21
	M9	43.55±0.70	40.02±0.65	26.79±0.87	26.81±.02
Hexyl acetate	M1	0.30±0.02	0.25±0.01	0.12±0.01	0.10±0.01

	M2	0.22±0.01	0.18±0.01	N.d.	0.02±0.01
	M3	0.15±0.02	0.20±0.02	0.12±0.06	N.d.
	M4	0.34±0.01	0.29±0.01	0.27±0.03	0.20±0.04
	M5	0.24±0.01	0.25±0.01	N.d.	N.d.
	M6	0.28±0.01	0.23±0.01	N.d.	N.d.
	M7	0.13±0.01	0.10±0.01	N.d.	N.d.
	M8	0.24±0.02	0.20±0.02	0.05±0.02	N.d.
	M9	0.08±0.01	0.06±0.01	N.d.	N.d.
Hexanol	M1	0.76±0.01	0.66±0.01	0.64±0.40	0.60±0.35
	M2	0.67±0.02	0.65±0.02	0.54±0.02	0.58±0.10
	M3	0.51±0.05	0.52±0.04	0.36±0.11	0.39±0.08
	M4	0.97±0.01	0.91±0.01	0.79±0.03	0.90±0.06
	M5	1.10±0.10	1.10±0.12	0.85±0.14	0.95±0.15
	M6	0.99±0.12	0.87±0.11	0.68±0.10	0.72±0.16
	M7	0.45±0.06	0.45±0.06	0.29±0.10	0.36±0.10
	M8	0.57±0.05	0.51±0.04	0.43±0.05	0.50±0.06
	M9	0.70±0.08	0.50±0.08	0.46±0.07	0.38±0.09
(Z)-3-Hexenol	M1	1.91±0.10	1.72±0.05	1.46±0.12	1.44±0.12
	M2	1.79±0.16	1.61±0.15	0.74±0.05	0.86±0.05
	M3	2.77±0.13	2.51±0.11	2.17±0.16	1.88±0.18
	M4	2.00±0.05	1.80±0.05	1.53±0.14	1.46±0.17
	M5	1.89±0.16	1.80±0.14	1.19±0.14	1.15±0.21
	M6	2.56±0.18	2.32±0.15	1.45±0.22	1.49±0.24
	M7	0.31±0.03	0.25±0.03	0.19±0.03	0.19±0.02
	M8	0.49±0.04	0.33±0.13	0.11±0.04	0.10±0.04
	M9	0.95±0.05	0.94±0.04	0.71±0.06	0.74±0.07
1-Octen-3-ol	M1	N.d.	N.d.	N.d.	N.d.
	M2	N.d.	N.d.	N.d.	N.d.
	M3	N.d.	N.d.	N.d.	N.d.
	M4	0.03±0.01	N.d.	N.d.	0.03±0.01
	M5	0.02±0.01	0.02±0.01	N.d.	N.d.
	M6	0.06±0.01	0.06±0.02	N.d.	N.d.
	M7	0.06±0.01	0.02±0.01	N.d.	N.d.
	M8	N.d.	N.d.	N.d.	N.d.
	M9	0.03±0.01	0.05±0.02	N.d.	0.04±0.02

(E)-2-Pentenal	M1	0.06±0.01	N.d.	N.d.	N.d.
	M2	0.29±0.04	0.29±0.05	0.07±0.02	N.d.
	M3	0.32±0.01	0.28±0.01	0.15±0.04	0.12±0.04
	M4	0.14±0.01	0.10±0.01	N.d.	N.d.
	M5	0.22±0.01	0.23±0.04	N.d.	N.d.
	M6	0.11±0.03	N.d.	0.06±0.01	N.d.
	M7	0.04±0.01	N.d.	N.d.	N.d.
	M8	N.d.	N.d.	N.d.	N.d.
	M9	0.25±0.06	0.21±0.03	0.22±0.02	0.23±0.05
(E)-2-Hexenal	M1	3.02±0.04	2.90±0.04	1.97±0.06	2.07±0.11
	M2	8.46±0.01	8.01±0.01	3.30±0.02	3.30±0.06
	M3	4.72±0.07	4.50±0.07	2.40±0.12	2.40±0.15
	M4	7.50±0.12	7.11±0.11	4.52±0.14	4.40±0.23
	M5	0.92±0.01	0.92±0.01	0.62±0.08	0.62±0.07
	M6	11.21±0.45	10.58±0.43	5.50±0.29	5.48±0.15
	M7	0.79±0.01	0.77±0.01	0.75±0.03	0.76±0.04
	M8	0.85±0.06	0.86±0.06	0.42±0.10	0.44±0.12
	M9	1.26±0.10	1.24±0.10	1.00±0.11	1.04±0.10
(Z)-3-Hexenyl acetate	M1	2.22±0.02	2.02±0.02	1.53±0.10	1.51±0.12
	M2	2.06±0.01	2.09±0.01	1.86±0.03	1.90±0.06
	M3	2.14±0.01	1.91±0.01	1.75±0.17	1.75±0.15
	M4	1.90±0.04	1.69±0.04	1.40±0.08	1.35±0.11
	M5	2.75±0.01	2.77±0.01	2.10±0.06	2.11±0.05
	M6	1.35±0.01	1.13±0.01	0.90±0.06	0.90±0.04
	M7	0.02±0.01	N.d.	N.d.	N.d.
	M8	0.12±0.03	0.01±0.01	0.08±0.03	0.09±0.04
	M9	0.09±0.04	N.d.	N.d.	N.d.
6-Methyl-5-hepten-2-one	M1	N.d.	N.d.	N.d.	N.d.
	M2	N.d.	N.d.	N.d.	N.d.
	M3	N.d.	N.d.	N.d.	N.d.
	M4	N.d.	N.d.	N.d.	N.d.
	M5	N.d.	N.d.	N.d.	N.d.
	M6	N.d.	N.d.	0.07±0.02	0.06±0.02
	M7	0.35±0.01	0.34±0.01	0.22±0.01	0.18±0.02
	M8	0. 28±0.01	0.30±0.03	0.20±0.02	0.18±0.01

	M9	2.34±0.14	2.30±0.13	2.02±0.16	2.02±0.19
(E)-2-Hexenol	M1	0.18±0.01	0.11±0.01	N.d.	0.02±0.01
	M2	0.04±0.01	0.04±0.04	N.d.	N.d.
	M3	N.d.	N.d.	N.d.	N.d.
	M4	0.80±0.02	0.76±0.01	0.68±0.02	0.63±0.04
	M5	0.20±0.02	0.20±0.02	0.15±0.03	N.d.
	M6	0.46±0.02	0.40±0.02	0.40±0.08	0.40±0.07
	M7	1.89±0.03	1.86±0.02	1.64±0.07	1.65±0.09
	M8	N.d.	N.d.	N.d.	N.d.
	M9	0.10±0.01	0.09±0.01	N.d.	0.03±0.01
Acetic acid	M1	20.50±0.74	19.34±0.70	6.90±0.84	6.21±0.69
	M2	1.14±0.77	1.05±0.73	7.99±0.30	8.01±0.38
	M3	0.18±0.09	0.20±0.08	1.35±0.34	1.35±0.37
	M4	5.78±0.72	5.37±0.68	3.08±0.41	3.00±0.46
	M5	2.21±0.58	1.98±0.55	1.46±0.29	1.46±0.48
	M6	0.57±0.04	0.42±0.04	1.99±0.83	1.99±0.78
	M7	0.81±0.03	0.65±0.03	0.95±0.24	0.96±0.31
	M8	6.33±1.26	5.90±1.20	2.75±1.49	2.81±1.51
	M9	10.03±1.31	9.41±1.25	10.73±1.32	11.19±1.44

Note: N.d.: Compound not detected. SD: standard deviation. EC: Quantification performed by external standard calibration. EC with IS: Quantification performed by external standard with internal standard calibration. AC: Quantification performed by standard addition calibration. AC with IS: Quantification performed by standard addition with internal standard calibration.
